# Second-line erlotinib after failure of pemetrexed-containing chemotherapy in advanced non-small cell lung cancer (NSCLC): Real-world effectiveness, safety and tolerability

**DOI:** 10.1371/journal.pone.0215135

**Published:** 2019-04-11

**Authors:** Paul Germonpré, Tim Van den Wyngaert

**Affiliations:** 1 Faculty of Medicine and Health Sciences, University of Antwerp, Wilrijk, Belgium; 2 Department of Pneumology, AZ Maria Middelares, Ghent, Belgium; 3 Department of Nuclear Medicine, Antwerp University Hospital, Edegem, Belgium; Istituto di Ricovero e Cura a Carattere Scientifico Centro di Riferimento Oncologico della Basilicata, ITALY

## Abstract

**Introduction:**

Little data is available on patients with advanced non-squamous NSCLC treated with erlotinib specifically after failure of first-line pemetrexed-containing chemotherapy. We assessed the effectiveness, safety and tolerability of erlotinib in a real-world setting.

**Methods:**

Prospective single-arm, open-label, multicenter, non-interventional study of erlotinib (150mg daily) in inoperable stage III/IV NSCLC after progression on first-line pemetrexed-containing chemotherapy without EGFR-mutation selection. Patients were followed according to routine practice and response assessment was performed using RECIST 1.1. The primary end point was progression-free survival (PFS). Secondary end points included best confirmed overall response rate (ORR), disease control rate (DCR), and overall survival (OS). Adverse events were recorded. An independent dataset was used to validate the results.

**Results:**

In all, 59 patients were screened, 57 enrolled, and 54 (36 men; median age 65 years) included in the per-protocol analysis. Median PFS was 1.8 (95% CI 1.4–2.6) months, with 11% (95% CI 5–21%) alive and progression-free at 6 months. The ORR was 0.0% (97.5% CI 0.0–6.8%) and the DCR 34.6% (95% CI 21.9–49.0%). Median overall survival was 5.8 (95% CI 3.3–8.6) months with 28% (95% CI 17–42%) alive at one year. Rash occurred in 60.7% (95% CI 46.7–73.5%), with severe rash in 12.5% (95% CI 5.1–24.1%). Any grade diarrhea was observed in 42.8% (95% CI 29.7–56.8%), with grade 3 occurring in 7.1% (95% CI 1.9–17.2%). Erlotinib was stopped in 21.0% (95% CI 11.3–33.9%) of patients due to adverse events, which were treatment related in 7%.

**Conclusion:**

Second-line erlotinib after pemetrexed treatment results in similar real-world outcomes as reported after non-pemetrexed containing first-line therapy. However, the overall duration of response in unselected patients remains limited and other effective treatments have in the meantime been introduced. No new safety signals were detected.

## Introduction

With an estimated 279.400 deaths in Europe in 2015, lung cancer remains the most frequent and lethal cancer in men and has equaled breast cancer mortality in woman.[1] Treatment options for non-small cell lung cancer (NSCLC), which accounts for approximately 80% of all lung cancers, have increased considerably over the last decade, yet it still remains a largely fatal disease. In addition, the benefit of most novel treatment options is conditional on the presence of specific tumor biomarkers associated with response (e.g. expression of programmed death ligand 1 [PD-L1], or particular gene mutations or rearrangements).[2] Erlotinib is an oral highly potent reversible tyrosine kinase inhibitor (TKI) that targets the Human Epidermal Growth Factor Receptor Type 1 (HER1)/Epidermal Growth Factor Receptor (EGFR). In Europe, erlotinib is approved for the first-line treatment of patients with locally advanced or metastatic NSCLC with EGFR activating mutations, for switch maintenance treatment in patients with locally advanced or metastatic NSCLC with EGFR activating mutations and stable disease after first-line chemotherapy. Also, erlotinib is indicated for the treatment of patients with locally advanced or metastatic NSCLC after failure of at least one prior chemotherapy regimen: if a positive EGFR-expression is demonstrated on immunohistochemistry, or in disease without EGFR activating mutations if other treatment options are not considered suitable.[3]

The approval of erlotinib as second-line therapy is based on the pivotal double-blind BR.21 trial in which 731 patients with stage IIIB/IV NSCLC, who had failed at least one previous chemotherapy regimen and were not eligible for further chemotherapy, were randomized to receive erlotinib (*n* = 488) or placebo (*n* = 243). In this study, erlotinib prolonged progression-free survival (PFS) (median 2.2 versus 1.8 months; HR 0.61; *P*<0.001) and overall survival (OS) (median 6.7 versus 4.7 months; HR 0.70; *P*<0.001).[4] Only 5% of patients discontinued erlotinib because of toxic effects, demonstrating the safety and tolerability of this treatment in these patients. In addition, erlotinib improved tumor-related symptoms and important aspects of quality of life.[5] These findings were subsequently confirmed in a global phase IV study (TRUST) with over 6,000 patients, including 3,224 patients receiving second-line erlotinib treatment.[6, 7]

It has been reported that the choice of first-line therapy can impact the effectiveness of subsequent treatments and evolving treatment options in locally advanced or metastatic NSCLC have led to knowledge-gaps regarding the sequencing of specific therapeutic agents.[8] For example, the parallel clinical trials leading to the approval of erlotinib (2005) and pemetrexed (2004) resulted in a paucity on outcome data of patients treated with erlotinib after failure of first-line pemetrexed, even within reported post-marketing authorization phase IV study results.[4, 9]

In the TIME study (“Tarceva as second-line treatment after pemetrexed in advanced NSCLC”), we aimed to address this issue and assess the effectiveness, safety and tolerability of erlotinib as second-line treatment in EGFR mutation unselected patients with advanced non-squamous NSCLC after failure of a first-line pemetrexed-containing chemotherapy regimen.

## Methods

### Patients

This study recruited patients between September 2011 and October 2012, with the first registered patient assessment on 2 September 2011 and the last patient visit on 22 August 2013. Patients with histologically or cytologically documented inoperable stage III or stage IV NSCLC who progressed after first-line treatment with a pemetrexed-containing chemotherapy regimen and were candidate for second-line treatment with erlotinib were eligible. Additional inclusion criteria included: age 18 years or older, Eastern Cooperative Oncology Group (ECOG) performance status (PS) 0–2, and written informed consent. As required by the federal Belgian regulatory authorities, EGFR expression in at least 10% of tumor cells as determined with immunohistochemistry was mandatory to be eligible for erlotinib, even though solid data backing this requirement are lacking.[10, 11] Patients who received other chemotherapy or targeted therapy after disease progression during first-line treatment, or who had any contra-indication for erlotinib (including hypersensitivity) were excluded. The study was approved by the institutional review board of all participating centers.

### Design

The TIME study was a prospective single-arm, open-label, multicenter, non-interventional phase IV trial conducted in Belgium in a real-world clinical setting. After screening and informed consent, erlotinib treatment was started in accordance with the summary of product characteristics (SPC), with a recommended daily dose of 150 mg.[3] Treatment modifications were allowed in accordance to the SPC, and erlotinib treatment was stopped in case of disease progression. Any temporary treatment interruption was recorded but was not considered as treatment stop if erlotinib was restarted and no other anticancer therapy had been used during the interruption. Concomitant use of erlotinib with chemotherapy was not allowed. Recorded baseline variables included: demographics, disease characteristics, and first-line treatment details. Clinical follow-up and treatment assessments during erlotinib treatment were according to routine institutional clinical practice. There were no required tests, examinations, or other observations and assessments. Collected data included: erlotinib treatment details, tumor response assessments, and adverse events (during treatment or up to 1 month after stop of erlotinib). Study participation ended in case of death, withdrawal of consent, lost-to-follow-up or study termination, whichever occurs first. The trial was registered on ClinicalTrials.gov: NCT01664533.

### End points

The primary end point of this study was progression-free survival (PFS) as assessed using the Response Evaluation Criteria in Solid Tumors (RECIST) version 1.1.[12] Secondary end points included: best confirmed overall response rate (ORR), disease control rate (DCR), and overall survival (OS). Any complete response (CR) or partial response (PR) had to be “confirmed” by a subsequent RECIST evaluation that also showed a CR or PR in order to reduce possible bias caused by differences in follow-up. The ORR was defined as the proportion of patients who achieved confirmed CR or PR during therapy. DCR consisted of patients with stable disease (SD), PR or CR as best overall confirmed response. If RECIST evaluation was not possible, non-quantitative criteria or clinical criteria (like palpation of superficial lymph nodes, or appearance of visible cutaneous metastasis) could be used as surrogate markers of disease progression, but not of response. Finally, safety and tolerability were assessed by analysis of reported adverse events, with rash and diarrhea being adverse events of interest. Rash was graded as mild, moderate, or severe using a published classification system and the highest scores per patient during treatment are reported.[13] Likewise, standardized assessment of diarrhea was performed on a 5 point scale using the Common Terminology Criteria for Adverse Events (CTCAE) version 4.[14]

### External validation

To validate the outcomes of this single-arm study, individual patient-level data from Project Data Sphere were used.[15] This repository contains deidentified comparator-arm data from completed oncology trials and was queried for studies with erlotinib in locally advanced or metastatic NSCLC in a second-line setting. Two such trials were identified: 1) A6181087: comparing the combination of sunitinib and erlotinib with placebo and erlotinib (NCT00457392) [16], and 2) ZEST: studying vandetanib versus erlotinib (NCT00364351) [17]. Baseline characteristics (gender, age, race, smoking history, ECOG status, and disease stage), prior anticancer treatments, PFS during erlotinib treatment, and MedDRA coded adverse events were extracted for 460 and 512 patients in the A6181087 and ZEST trials, respectively. Extracted time-to-event data were compared with published Kaplan-Meier curves to validate the data extraction process. Patients with first-line pemetrexed treatment (A6181087: *n* = 46; ZEST: *n* = 53) were selected and a pooled analysis of PFS was conducted. Adverse events reported in these patients were screened for skin toxicity and diarrhea related MedDRA codes.

### Statistical methods

A convenience sample size of 200 patients was estimated based on the expected accrual potential during 2 years. Given the real-world setting of this study, there was no formal predefined statistical hypothesis. The per-protocol population consisted of all enrolled patients who started erlotinib therapy and did not violate the study protocol while on study. The safety analysis population was defined as all patients with at least one intake of erlotinib, regardless of whether they completed the study protocol.

For survival end-points, the time in months from the start of erlotinib was used. Censoring for PFS was applied for patients who stopped erlotinib, without progression or death under erlotinib at the study termination date. For patients who terminated the study under erlotinib, without progression or death under erlotinib, and for patients who were ongoing in the study under erlotinib, without stopping erlotinib, progression or death under erlotinib, censoring occurred at the date of study termination or the date of last visit, respectively. Censoring for OS was applied for patients whose date of death was not documented. Time-to-event data were analyzed using the Kaplan-Meier method and Cox proportional-hazards regression.[18] Hazard ratios are reported with accompanying 95% confidence intervals (CI). Model diagnostics were performed as appropriate, including interaction testing and analysis of residuals.[19] For proportion outcomes, exact binomial 95% confidence intervals are reported. In case of a numerator of zero, an exact binomial one-sided 97.5% confidence interval is used instead.

Planned subgroup analyses of effectiveness according to baseline characteristics included: disease stage, best response after first-line therapy, ECOG status, gender, presence of brain metastases, smoking status, age of 60 years or older, and development of rash during erlotinib treatment. All analyses were performed using Stata 13.0 (StataCorp, College Station, TX, USA). The trial was registered on ClinicalTrials.gov with identifier NCT01664533.

## Results

### Patient disposition

**Fig 1:**
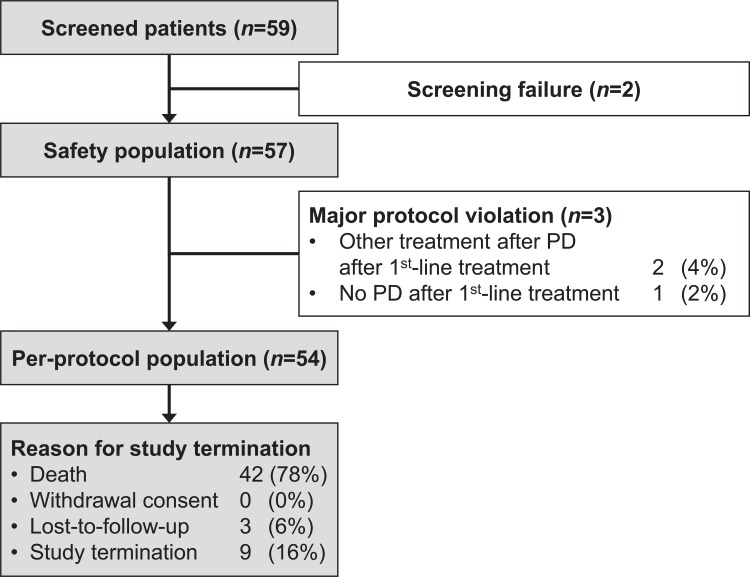
CONSORT diagram of patients.

A total of 59 patients were screened at 17 specialist oncology and pneumology centers in Belgium, of which 57 started erlotinib therapy and were included in the safety analysis (Fig 1). The trial was stopped prematurely due to a slower than expected patient accrual. The last patient visit occurred on 22 August 2013. The median duration of follow-up was 7.4 (95% CI 5.7–9.0) months and the median interval between data capture visits from the patients’ medical record was 30.5 days (interquartile range 46). Three patients had major protocol violations and were excluded, yielding a per-protocol study population of 54 patients. The main reason for study discontinuation was death (*n* = 42; 77.8%), and less frequently study termination (*n* = 9; 16.7%), or lost-to-follow-up (*n* = 3; 5.6%). Baseline patient characteristics are summarized in Table 1. Only one patient had an activating EGFR mutation, out of 42 sampled. First-line chemotherapy consisted of a median of 4 (range 1–13) cycles of a pemetrexed-based regimen, with pemetrexed dosed at 500mg/m² in nearly all patients (*n* = 50; 92.6%). This was combined with cisplatin in all except 5 patients (90.7%), with 75mg/m² being the most common dose (91.8%). Most patients (83.3%) did not require a change in chemotherapy regimen in subsequent cycles, but cisplatin was omitted during the course of first-line treatment in 6 (11.1%) of patients.

**Table 1:** Baseline characteristics of the patients in the per-protocol population (*n* = 54).

Characteristic	Median (range) / N (%)
Age (years)	65 (43–79)
Gender	
Male	36 (66.7%)
Female	18 (33.3%)
Ethnic group^a^	
European descent	54 (100%)
Smoking status	
Non-smoker	7 (13.0%)
Current of ever smoker	47 (87.0%)
Performance status (% of patients)	
0	14 (25.9%)
1	28 (51.9%)
2	12 (22.2%)
Time since diagnosis at start erlotinib (months)	6.8 (1.4–33.7)
Disease stage (% of patients)	
IIIB	5 (9.3%)
IV	49 (90.7%)
Histology	
Adenocarcinoma	53 (98.1%)
Other/Unknown	1 (1.9%)
Activating EGFR mutation (available samples *n* = 42)	
Positive	1 (2.4%)
Negative	41 (97.6%)
Brain metastases (data available *n* = 49)	
Yes	10 (20.4%)
No	39 (79.6%)
Platinum-based 1^st^-line chemotherapy	
Yes	49 (90.7%)
No	5 (9.3%)
Response to 1^st^-line chemotherapy	
Complete or partial response	26 (48.1%)
Stable disease	7 (13.0%)
Progressive disease	21 (38.9%)

^a^ Self-reported or investigator determined, not based on country of domicile.

### Effectiveness

**Fig 2:**
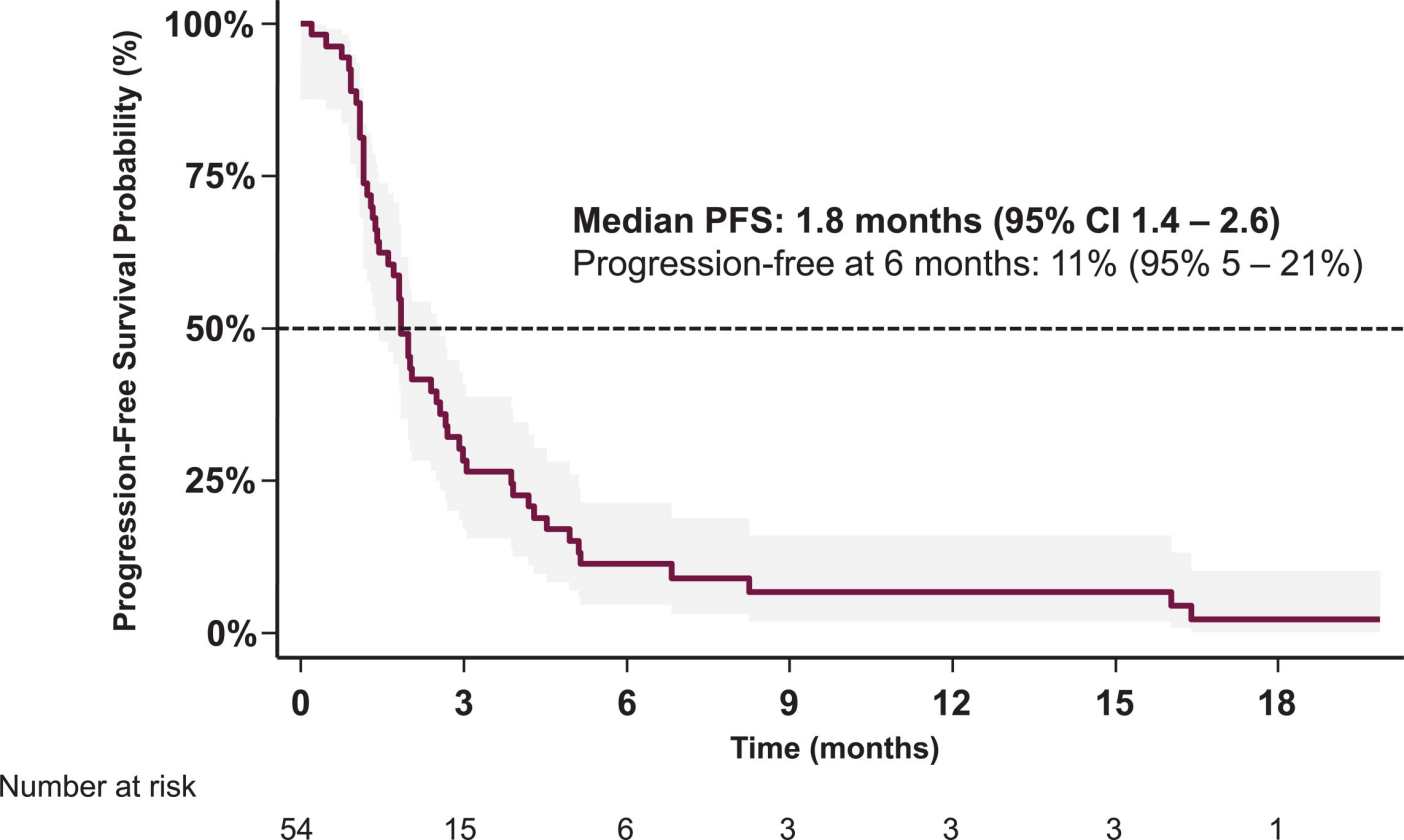
Progression-free survival (PFS) during erlotinib therapy (with 95% CI).

**Fig 3:**
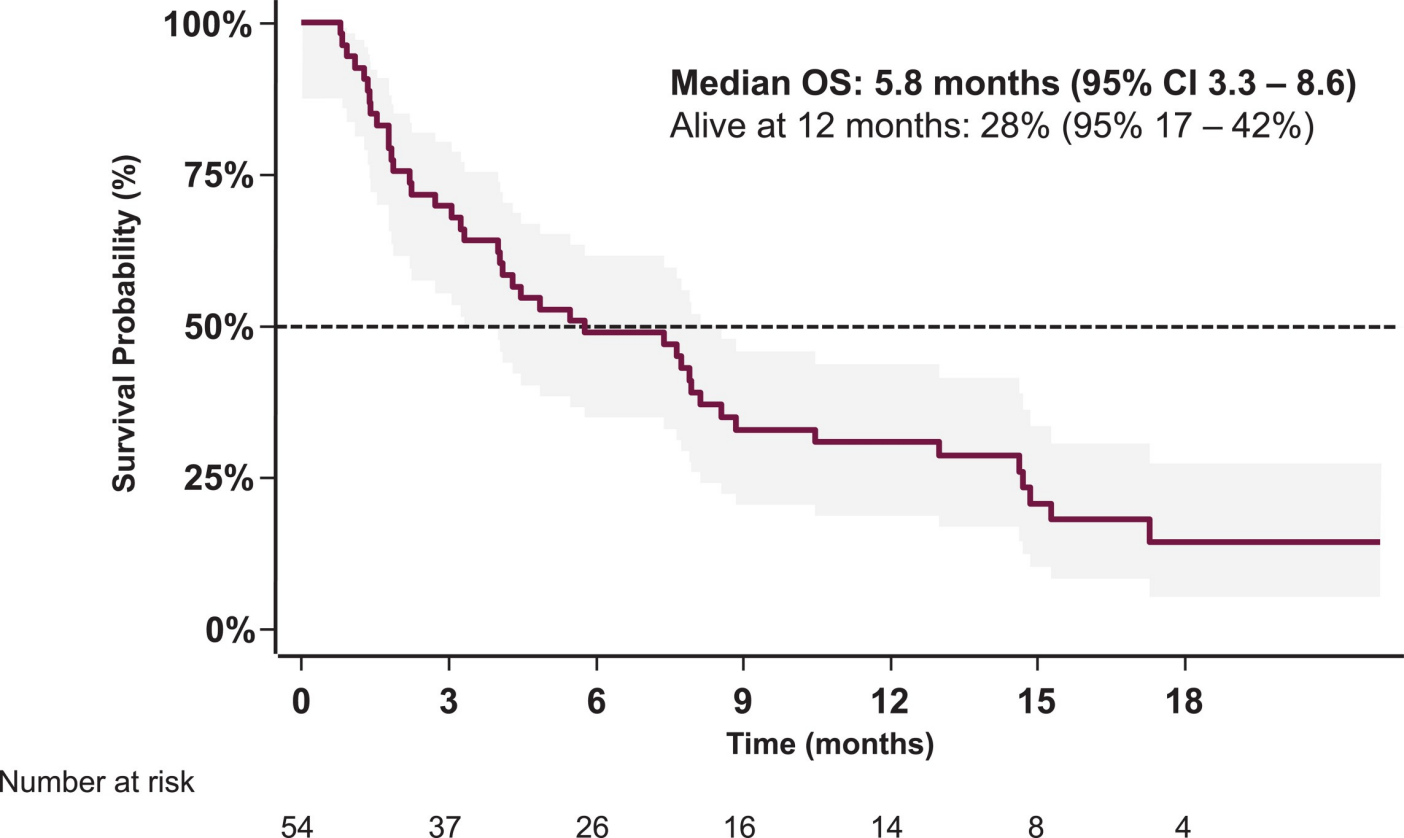
Overall survival (OS) of the study population (with 95% CI).

At database lock, 42 (77.8%) deaths had occurred and 34 (63.0%) disease progression events were documented under erlotinib. The mean number of radiological tumor response assessments per patient after baseline scanning was 1.3 (95% CI 0.88–1.83). The median PFS during erlotinib treatment was 1.8 (95% CI 1.4–2.6) months, with a 6 month progression-free survival rate of 11% (95% CI 5–21%) (Fig 2). There were no confirmed complete or partial responses under second-line erlotinib (ORR 0.0%; 97.5% CI 0.0–6.8%), with assessments available for 52 patients. One third of patients had stable disease, yielding a DCR of 34.6% (95% CI 21.9–49.0%). In 65.4% (95% CI 50.9–78.0%) of patients, progressive disease was the best documented confirmed response. The median overall survival was 5.8 (95% CI 3.3–8.6) months and one year after starting erlotinib 28% (95% CI 17–42%) of patients were still alive (Fig 3).

### Safety and toxicity

A dose reduction in erlotinib treatment was recorded in 7 out of 57 evaluable patients (12.3%). Erlotinib was interrupted in 8 patients for on average 8.8 days (range 3–23). The reason for erlotinib cessation was disease progression (75.5%), death (11.3%), intolerability (7.5%), or for unknown reasons in 5.7% of patients. Out of 56 evaluable patients, 34 (60.7%; 95% CI 46.7–73.5%) developed rash during erlotinib treatment. Severe rash was very common (12.5%; 95% CI 5.1–24.1%), with mild or moderate rash occurring in 17.8% (95% CI 8.9–30.3%) and 30.4% (95% CI 18.7–44.0%), respectively. Diarrhea occurred in 24 out of 56 evaluable patients (42.8%; 95% CI 29.7–56.8%), with grade 1 in 28.5% (95% CI 17.2–42.2%), grade 2 in 7.1% (95% CI 1.9–17.2%), and grade 3 occurring in 7.1% of patients (95% CI 1.9–17.2%). Other adverse events were reported in 51 (89.4%) patients of the safety population (*n* = 57) (Table 2). Erlotinib was stopped in 12 (21.0%; 95% CI 11.3–33.9%) patients due to adverse events, but only in 4 patients were these assessed as related to erlotinib use: vomiting, skin ulcer, decreased appetite, and renal failure.

**Table 2:** Ten most frequently reported adverse events.

Adverse event^a^	Count/% (*n* = 57)
Decreased appetite	13 (9.2%)
Weight decreased	8 (5.6%)
Asthenia	6 (4.2%)
Nausea	5 (3.5%)
Pyrexia	5 (3.5%)
Cough	5 (3.5%)
Anaemia	4 (2.8%)
Vomiting	4 (2.8%)
Fatigue	4 (2.8%)
General physical health deterioration	4 (2.8%)

^a^ Categories are not mutually exclusive.

### Subgroup analyses

**Fig 4:**
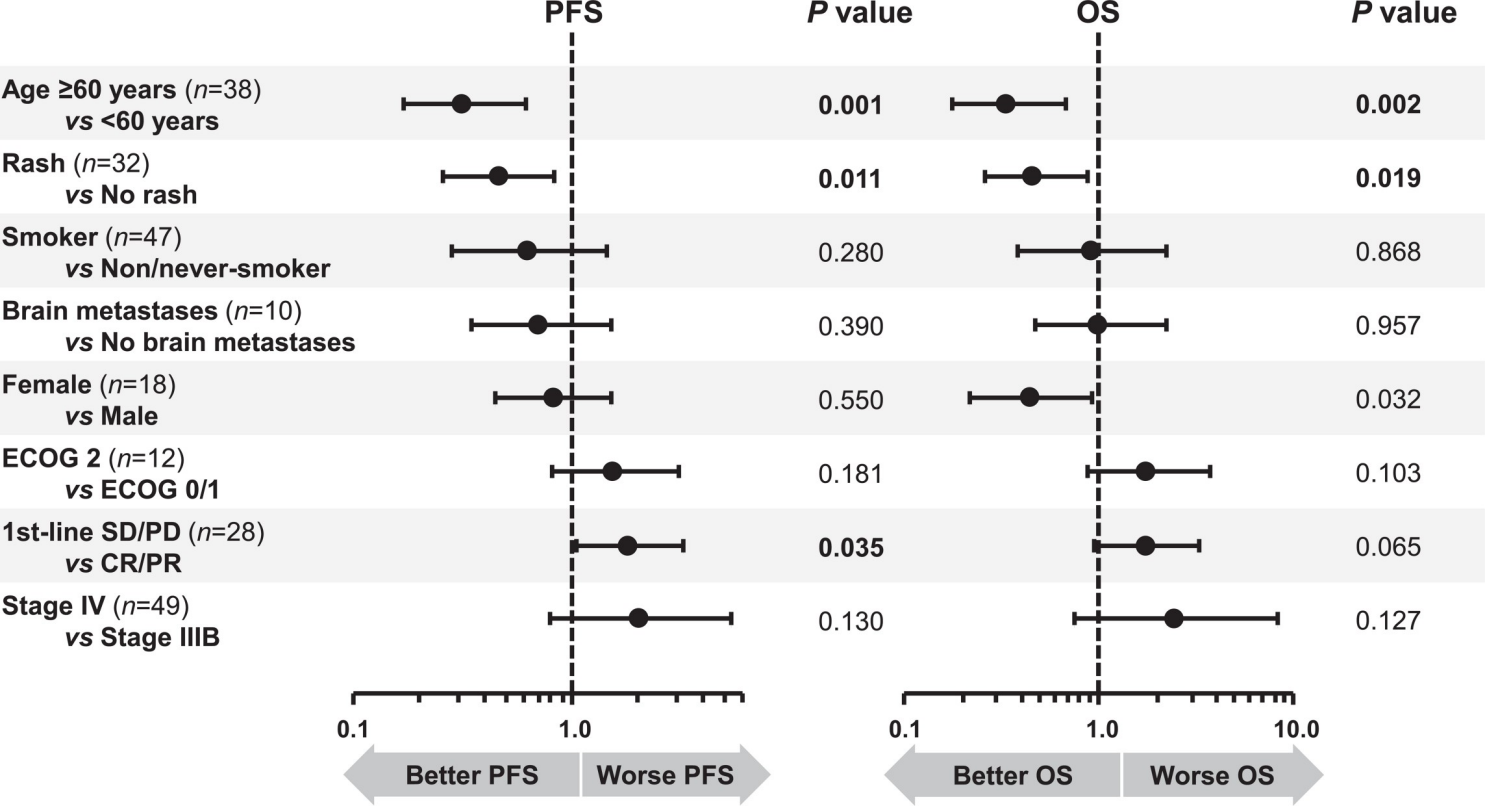
Forest plot of hazard ratios (HR) of baseline predictors of overall survival (OS).

Baseline patient characteristics associated with PFS and OS are summarized in Fig 4. In summary, patients aged 60 years or older had a significantly lower risk of death or disease progression (HR 0.32; 95% CI 0.17–0.61; *P =* 0.001) or of death (HR 0.34; 95% CI 0.17–0.67; *P =* 0.002). In a landmark analysis, patients who developed any grade of rash by the first follow-up data extraction visit had better PFS (HR 0.47; 95% CI 0.26–0.84; *P =* 0.011) and OS (HR 0.47; 95% CI 0.26–0.89; *P =* 0.019). Female patients had a better overall survival compared to males (HR 0.45; 95% CI 0.22–0.93; *P =* 0.03). In contrast, patients with SD/PD as best response to first-line chemotherapy had a higher chance of death or disease progression (HR 1.82; 95% CI 1.04–3.19; *P =* 0.03).

### External validation

Individual patient-level data were available for 99 patients (10.2%) from the control groups of the A6181087 and ZEST trials who received erlotinib after first-line pemetrexed-containing chemotherapy. Patient characteristics were comparable for gender (*P* = 1.000), age (*P* = 0.355), and smoking status *(P* = 0.560), but the TIME study population had a worse mean ECOG score (0.96 versus 0.65; *P* = 0.004) and only included subjects of European descent whereas 16% were of non-European descent in the validation set (*P* = 0.011). The median PFS was 1.9 (95% CI 1.8–2.6) months with broadly overlapping confidence intervals with that from the TIME study (1.8 months; 95% CI 1.4–2.6). Also, after controlling for differences in race and ECOG status there was no difference between both cohorts (HR 1.02; 95% CI 0.71–1.47). In the validation cohort, any grade rash or diarrhea occurred in 73.7% (95% CI 63.9–82.1%) and 40.4% (95% CI 30.6–50.7%) of patients during erlotinib treatment, respectively.

## Discussion

The expanding number of available therapies in locally advanced or metastatic NSCLC provides unique challenges, in particular with respect to outcome data on specific treatment sequences. The EGFR targeted tyrosine kinase inhibitors (TKI) erlotinib, afatinib or gefitinib are available as second-line treatments, although the latter two may only be available in some regions and limited to patients with a tumor bearing an activating EGFR mutation. These anti-EGFR TKIs have demonstrated a favorable hematologic toxicity profile (e.g. less grade 3–4 neutropenia and thrombocytopenia) compared to the chemotherapeutic alternatives and can provide a treatment alternative in patients in whom a break from chemotherapy is preferred.[4, 20, 21]

The TIME study specifically included patients who progressed after a first-line pemetrexed containing chemotherapy regimen, as there is very little data available on this particular treatment sequence due to the parallel clinical development of both agents. Otherwise, the patient characteristics were typical of that of a sample of advanced lung cancer patients, consisting mainly of men of European descent over the age of 60 who were active or former smokers. The median time-to-progression during first-line pemetrexed observed in TIME (4.5 months) was comparable with the results of the registration study with this agent (3.4 months).[9]

The PFS during erlotinib treatment in TIME (1.8 months; 95% CI 1.4–2.6) was nearly identical to that of the external validation cohort (1.9 months; 95% CI 1.8–2.6), supporting the validity of this finding. These results fall within the range reported in a number of previous studies that examined erlotinib in a second-line setting, even though not specifically after first-line pemetrexed treatment as in TIME (Table 3).

**Table 3:** Overview of available outcome data with erlotinib as a second-line treatment.

Study	Intervention	Patients (n)	Median PFS (months) (95% CI)	Median OS (months) (95% CI)
TIME	Erlotinib	57	1.8 (1.4–2.6)	5.8 (3.3–8.6)
BR.21[4]	Erlotinib *vs* placebo	731	2.2 (N/R)	6.7 (N/R)
TITAN[22]	Erlotinib *vs* docetaxel/pemetrexed	424	1.5 (1.4–1.6)	5.3 (4.0–6.0)
TRUST[7]	Erlotinib	3,224	3.1 (2.9–3.4)	8.6 (8.2–9.2)
HORG[23]	Erlotinib *vs* pemetrexed	357	3.6 (N/R)	8.2 (N/R)
TAILOR[24]	Erlotinib *vs* docetaxel	222	2.4 (2.1–2.6)	5.4 (4.5–6.8)

N/R: not reported.

The 95% confidence intervals of the observed PFS and OS in TIME are overlapping with the values reported in these studies, with PFS ranging from 1.5 to 3.6 months and OS between 5.3 and 8.6 months. However, this comparison is hampered by the small sample size of the TIME cohort compared to the other trials. Of note, concerns have surfaced on the validity of the results of the TAILOR study, but they are included here for completeness.[25] A study of erlotinib in second or third line setting (DELTA) reported a similar PFS of 2.0 (95% CI 1.3–2.8) months.[26] Studies with the EGFR inhibitor gefitinib as second or third line treatment showed comparable PFS outcomes of 2.0 (V-15-32) and 2.2 months (INTEREST).[20, 27] Taken together, first-line treatment with pemetrexed does not result in better or inferior outcomes with subsequent erlotinib treatment. However, the overall duration of response to EGFR inhibition in unselected patients remains limited, regardless of the agent used.

The start dose of erlotinib in TIME was the recommended reference dose of 150 mg in all patients and dose reductions during the study were only required in 12.3% of patients, which is in line with the 17% reported in the TRUST study.[7] Treatment discontinuations occurred in 21.0% of patients, as compared to 21.5% in BR.21.[4] Of note, most discontinuations in TIME were due to an adverse event not related to erlotinib, as only 4 (7.0%) patients stopped treatment because of unacceptable toxicity. No deaths were attributed to erlotinib use. Collectively these findings confirm the favorable tolerability of erlotinib.

In general, adverse events were frequent, as can be expected in a population of patients with advanced cancer. The majority of patients (60.7%) developed some degree of rash during erlotinib treatment. However, in the vast majority (79.4% of patients with rash) this was mostly of mild (= without associated symptoms) or moderate severity (= with pruritus or other associated symptoms covering <50% of body surface). Severe or disabling rash occurred in 12.5% of patients with rash. These results are comparable with the skin toxicity reported in the TRUST study, where 71% of patients developed rash, with 83% of mild or moderate severity and 17% had severe or disabling rash.[7] Similarly, in the validation cohort rash was documented in 73.7% of patients. The TIME data also confirm the prognostic value of developing skin rash during erlotinib treatment, which was associated with a risk reduction in disease progression or death of about 50%.[6]

Approximately 4 out of 10 patients (42.8%) reported some degree of diarrhea while receiving erlotinib. Grade 3 diarrhea was very common (7.1%) and out of all subjects with some diarrhea, this was scored as mild (i.e. an increase of <4 stools per day over baseline) or moderate (i.e. an increase of 4–6 stools per day over baseline) in 83.3% of patients. This finding is comparable with the results from previous phase III studies with erlotinib, documenting some degree of diarrhea in 18%[22], 30%[24], and 55%[4] of patients. Similar findings were observed in the validation cohort as well, with any grade diarrhea occurring in 40.4%. Ultimately, no new safety signals with respect to erlotinib were detected in the TIME study.

The TIME study had a number of weaknesses. First, the study was closed prematurely due to slow accrual with only 30% (n = 57) of the intended number of patients enrolled. As a result, the estimates obtained from this limited sample have broad confidence intervals and subsequent inferences are limited in accuracy or may lack power. Therefore, interpretation of the results should consider the reported 95% confidence intervals over the actual point estimates. To mediate some of these effects and provide context, an external validation cohort was compiled from the Project Data Sphere trial repository. This independent data set consisting of patients that closely match those of the TIME study was used to provide a benchmark for the reported outcome data. Moreover, only 10.2% of patients in these clinical trials were treated in first line with pemetrexed highlighting the unique patient population addressed in the TIME study. Secondly, data on EGFR mutational status was only available for 42 (77.8%) patients and no data was available on other tumor mutations (e.g. ALK, ROS1, BRAF) for which targeted treatments now exist. Also, patients were selected for erlotinib treatment based on EGFR expression according to local regulatory requirements rather than mutational status as currently advised.[28] The median PFS observed in TIME (1.8 months) supports that EGFR expression is unable to select patients who will derive greater benefit from erlotinib treatment. Indeed, recent data with second-line erlotinib treatment show a nearly identical median PFS as in TIME for EGFR wild-type patients (1.9 months), in stark contrast to those with EGFR-sensitizing mutations (10.2 months; *P*<0.001).[29]

Thirdly, no quality-of-life data were collected. Finally, the introduction of immunotherapy has drastically overhauled the treatment landscape of locally advanced or metastatic NSCLC. When used after progression on one or two platinum-based chemotherapy regimens both atezolizumab and nivolumab resulted in significant gains in median overall survival (nivolumab 12.2 months [95% CI 9.7–15.0]; atezolizumab 13.8 months [95% CI 11.8–15.7]) compared to docetaxel. Intriguingly, median progression-free survival was not improved with either agent (nivolumab 2.3 months [95% CI 2.2–3.3]); atezolizumab 2.8 months [95% CI 2.6–3.0]) and is similar to that reported with erlotinib.[30, 31] A recent Bayesian network meta-analysis of 102 randomized-controlled trials suggested that nivolumab, pembrolizumab, atezolizumab, and pemetrexed plus erlotinib may be the most effective second-line treatments for NSCLC in terms of OS.[32] Nevertheless, for patients who have known contra-indications for immunotherapies or subsequent chemotherapy after first-line treatment, the results from the TIME study may provide valuable information.

## Conclusions

Second-line erlotinib after progression on pemetrexed results in similar real-world outcomes as reported after non-pemetrexed containing first-line treatments. However, the overall duration of response in unselected patients remains limited, as observed with other EGFR targeting agents, and other effective treatments have in the meantime been introduced. Adverse events were similar to the established safety profile of erlotinib, with rash and diarrhea being very common, but mostly of mild or moderate severity.

## Supporting information

S1 File TIME dataset.txt contains the individual patient level data of the per-protocol analysis cohort of the TIME study. A detailed explanation of the variables and their interpretation is provided in the file.
(TXT)

S2 File 20120920 ML25708 protocol V2.pdf contains the full study protocol. (PDF)

S3 File trendstatement.pdf provides the completed TREND questionnaire for the TIME study. (PDF)
