# Supplementary material for: Second-line erlotinib after failure of pemetrexed-containing chemotherapy in advanced non-small cell lung cancer (NSCLC): Real-world effectiveness, safety and tolerability
Source: PLoS One. 2019 Apr 11;14(4):e0215135. doi: 10.1371/journal.pone.0215135 (PMC6459587; doi:10.1371/journal.pone.0215135)
Supplement: S2 File — (PDF) [file pone.0215135.s002.pdf]

NV ROCHE SA

Prospective, open-label, multicenter, national, non-interventional phase IV trial of the effectiveness, safety and tolerability of Tarceva® as second-line treatment of patients with advanced non-small cell lung cancer (NSCLC), after failure of first-line treatment with a pemetrexed-containing chemotherapy regimen

"*TIME* (Tarceva® as second-line treatment after pemetrexed in advanced NSCLC)"

A NON-INTERVENTIONAL STUDY

**Protocol Number:**	**ML25708**
Date of Final Protocol:	September 17, 2012
Protocol version:	version 2
Country Medical Manager:	Lieselot De Vos, PhD
Biostatistician: Matrix 45, LLC	Ivo Abraham
Pharmacovigilance	Dr. Christine Lenaerts

Confidentiality Statement

The information contained in this document, especially unpublished data, is the property of NV ROCHE SA (or under its control), and therefore provided to you in confidence as a potential participant or participant of this non-interventional study, potential investigator or consultant, for review by you, your staff and an applicable Independent Ethics Committee. It is understood that this information will not be disclosed to others without written authorization from ROCHE except to the extent necessary to obtain informed consent from those persons to whom the medication may be administered.

COUNTRY MEDICAL MANAGER

Lieselot De Vos, PhD
NV Roche SA
Dantestraat 75
1070 Brussel
BELGIUM
Email: lieselot.de_vos@roche.com
Tel: +32 2 525.82.58

STATISTICIAN

Ivo Abraham
Matrix 45, LLC
6159 West Sunset Road
Tuscon, Arizona, 85743
USA
Email: iabraham@matrix45.com, iabraham@email.arizona.edu
Tel: +1.303.997.2697, +1.202.487.3982

DATAMANAGEMENT

William Faria
Keyrus Biopharma
53 rue Baudin
92300 Levallois-Perret
France
Email: william.faria@keyrus.com
Tel: +33 1 41.34.28.28

PHARMACOVIGILANCE

Dr. Christine Lenaerts
NV Roche SA
Dantestraat 75
1070 Brussel
BELGIUM
Email: brussels.drug_safety@roche.com

Tel: +32 2 525.82.99

Fax: +32 2 525.84.66

COORDINATING PRINCIPAL INVESTIGATORS

Prof. Paul Germonpré
Dienst Longziekten
Universitair Ziekenhuis Antwerpen
Wilrijkstraat 10
2650 Edegem
BELGIUM
Email: paul.germonpre@uza.be
Tel: +32 3 821.34.47

Dr. Lionel Bosquée
Service Pneumologie
CHBAH Seraing
Rue Laplace 40
4100 Seraing
BELGIUM
Email: l.bosquee@chbah.be
Tel: +32 4.338.76.70

SYNOPSIS OF PROTOCOL

TITLE	Prospective, open-label, multicenter, national, non-interventional phase IV trial of the effectiveness, safety and tolerability of Tarceva® as second-line treatment of patients with advanced non-small cell lung cancer (NSCLC), after failure of first-line treatment with a pemetrexed-containing chemotherapy regimen<br>(TIME stands for Tarceva® as second-line treatment after pemetrexed in advanced NSCLC).
SPONSOR	NV Roche SA<br>Dantestraat 75<br>1070 Brussel<br>BELGIUM.
OBSERVED POPULATION	Patients with locally advanced or metastatic NSCLC (inoperable stage III or stage IV) who are treated with Tarceva® as second-line therapy and who have progressed after first-line treatment with a pemetrexed-containing chemotherapy regimen.
PRIMARY OBJECTIVE	▪ The progression-free survival (PFS) in patients with advanced stage III/IV NSCLC under Tarceva® as second-line therapy, after failure of a prior pemetrexed-containing chemotherapy regimen, will be described.
SECONDARY OBJECTIVES	▪ The best overall response, best confirmed overall response if applicable, response rate (RR) and disease-control rate (DCR) in patients with advanced stage III/IV NSCLC under Tarceva® as second-line therapy, after failure of a prior pemetrexed-containing chemotherapy regimen, will be described.<br>▪ The overall survival (OS) in patients with advanced stage III/IV NSCLC with Tarceva® as second-line therapy, after failure of a prior pemetrexed-containing chemotherapy regimen, will be described.<br>▪ Effectiveness data (PFS under Tarceva®, RR under Tarceva®, DCR under Tarceva® and OS from start of Tarceva® treatment) can be described and compared for different subgroups, depending on the size of the subgroups:<br>▪<br>- patients whose tumor response was assessed according to RECIST criteria vs patients whose tumor response was assessed according to other criteria.<br>- patients whose best overall response to 1L is complete response (CR) or partial response (PR) (at baseline) vs patients whose best overall response to 1L is stabile disease (SD) (at baseline) vs patients whose best overall response to 1L is progressive disease (PD) (at

	baseline).<br><br>- patients whose best overall response to 1L is CR or PR or SD (at baseline) vs patients whose best overall response to 1L is PD (at baseline).<br>- females (at baseline) vs males (at baseline).<br>- patients whose ECOG performance status (PS) is 0 or 1 (at baseline) vs patients whose ECOG PS is 2 (at baseline).<br>- patients whose ECOG PS is 0 (at baseline) vs patients whose ECOG PS is 1 (at baseline) vs patients whose ECOG PS is 2 (at baseline).<br>- patients with brain metastases (at baseline) vs patients with no brain metastases [(stage III cancer stage or bone metastases or liver metastases or adrenal gland metastases or other metastases at baseline) and (no brain metastases at baseline)].<br>- non-smokers (at baseline) vs former/current smokers (at baseline).<br>- patients with age <65 years (at baseline) vs patients with age ≥65 years (at baseline).<br>- patients with no rash during Tarceva® treatment vs patients with rash any toxicity (mild, moderate or severe) during Tarceva® treatment.<br>- supportive and additional evaluations may be conducted in other subgroup populations for exploratory reasons. These subgroups will be defined in the statistical analysis plan (SAP).<br>▪ Safety and tolerability of Tarceva® as second-line therapy, after failure of a prior pemetrexed-containing chemotherapy regimen, in patients with advanced stage III/IV NSCLC will be described [rash, diarrhea and other adverse events (AEs)].
STUDY DESIGN	Prospective, open-label, multicenter, national, non-interventional trial with Tarceva® monotherapy as second-line treatment after failure of first-line treatment with a pemetrexed-containing chemotherapy regimen.
NUMBER OF PATIENTS	Approximately 55.
NUMBER OF CENTERS	Approximately 20.
MAIN SELECTION CRITERIA	<u>Inclusion criteria:</u><br>• Signed written informed consent.<br>• ≥18 years.<br>• Histologically or cytologically documented diagnosis of locally advanced or metastatic NSCLC (inoperable stage III or IV according to 7th TNM classification).<br>• ECOG PS of 0-2.<br>• Experiencing PD after pemetrexed-containing

	chemotherapy regimen as 1L chemotherapy.<br>• Patient who started treatment with Tarceva® as 2L therapy, at the most 4 weeks prior to study entry at baseline (date of signature of informed consent).<br><u>Exclusion criteria:</u><br>• Received already other chemotherapy/targeted therapy treatment after PD after 1L treatment in advanced NSCLC setting.<br>• Contra-indication as in SmPC of Tarceva®: Hypersensitivity to Tarceva® or to any of the excipients.
LENGTH OF INDIVIDUAL OBSERVATION	Until death, withdrawal of consent, lost-to-follow-up or study termination (whichever occurs first, ~1 year).
DURATION OF STUDY	Recruitment stops on September 30, 2012 (estimated to start recruitment in Jul 2011).<br>The study is terminated at one year after the last patient in, or when all enrolled patients have ended the study, whichever occurs first.
CLINICAL AND LABORATORY OBSERVATIONS	<u>Effectiveness:</u><br>▪ Assessment of PFS, best overall response, best confirmed overall response if applicable, RR and DCR under Tarceva® treatment, by tumor assessment according to routine institutional clinical practice: radiographic assessment [through RECIST criteria (version 1.1) or through other interpretation criteria (non-standardized, non-quantitative criteria)] or other assessment (e.g. clinical criteria).<br>▪ Assessment of OS by date of death.<br><u>Safety:</u><br>▪ Assessment of safety and tolerability of Tarceva® (rash, diarrhea and other AEs):<br>➢ *On the CRF (clinical database):*<br>- Non-serious AEs (other than rash, diarrhea and PD after Tarceva®) are documented on an AE page of the CRF.<br>- Rash is documented on a separate section of the CRF.<br>- Diarrhea is documented on a separate section of the CRF.<br>- Serious AEs (SAEs) (other than rash, diarrhea, PD after Tarceva® and death) are documented on an AE page of the CRF.<br>- PD after Tarceva® is documented on a PD section of the CRF.<br>- Death is documented on a study end section of the CRF.

	➢ *Reporting to ROCHE (safety database):*<br>- Non-serious AEs (except for rash, diarrhea and PD after Tarceva®, for which the data from the clinical database will be transferred to the safety database at the termination of the study) are documented on an AE page of the CRF, will be collected by a monitor and forwarded to ROCHE.<br>- SAEs (except for PD after Tarceva® and death due to PD, for which the data from the clinical database will be transferred to the safety database at the termination of the study) must be reported by the participating physician or designee to ROCHE within 24 hours after becoming aware of the SAE. The SAE is reported by the site by means of fax, email or phone but preferably using the AE Reporting Form.
STATISTICAL ANALYSES	- The PFS and OS in the observed population will be described.<br>These variables will be analysed by the Kaplan-Meier method. Kaplan-Meier estimates for the survival rate and a Kaplan-Meier curve for survival will be presented. If possible the median survival time will be reported.<br>- The best overall response under Tarceva®, best confirmed overall response under Tarceva® in case of a best overall response of CR or PR, RR under Tarceva® and DCR under Tarceva® in the observed population will be described through descriptive statistics.<br>- Best overall response under Tarceva® is defined as the best tumor assessment to Tarceva® treatment, with hierarchy of CR>PR>SD>PD.<br>- Best confirmed overall response under Tarceva® will be described in case of a best overall response of CR or PR. Best confirmed overall response is defined as CR if at least two tumor assessments of CR have been made during Tarceva® treatment. Best confirmed<br>overall response is defined as PR, either if one tumor assessment of CR and at least one of PR have been made, or if at least two tumor assessments of PR have been made during Tarceva® treatment. Best confirmed overall response is defined as SD, if the definitions above of best confirmed overall response of CR or PR do not apply and at least one tumor assessment of SD has been made during Tarceva® treatment. Best confirmed overall response is defined as PD, if the definitions

above of best confirmed overall response of CR or PR or SD do not apply and at least one tumor assessment of PD has been made during Tarceva® treatment.

- RR under Tarceva® is defined as the rate of CR or PR as best overall response under Tarceva®.

- DCR under Tarceva® is defined as the rate of CR or PR or SD as best overall response under Tarceva®.

- Subgroup analyses (as explained in the secondary objectives) can be made to describe possible relationships between baseline and other assessments and the outcomes PFS, OS, RR and DCR, depending on the size of the subgroups.

- The subgroup analyses on time-to-event variables (PFS and OS) will be graphically presented by Kaplan-Meier curves, if possible, where p-values will be the results of a Log-Rank test and Wilcoxon test. In case both test results differ substantially, the Log-Rank test will be used for interpretation. The results of these tests can be further investigated by Cox Proportional Hazards models (CPH), to give a quantitative measurement for difference, such as a hazard ratio (HR) and its 95% confidence limits. A p-value for the test of statistical significance of that HR will be presented. The underlying assumption on proportional hazards of the CPH model will be tested.

- The subgroup analyses for binary data (RR and DCR) will be performed using a logistic regression model, if possible, resulting in odd's ratio (OR) estimates with their 95% confidence limits. A p-value for statistical significance of this OR will be presented.

- Safety and tolerability of Tarceva® in 2L will be described through descriptive statistics:

- AEs will be analysed patient-based as a categorical variable, with categories: treatment-emergent AEs, post-treatment AEs.

- SAEs will be analysed patient-based as a categorical variable.

- Deaths will be analysed patient-based as a categorical variable.

- Rash will be analysed patient-based as a categorical variable, with categories: No rash occurred, Rash occurred with worst severity: mild toxicity, Rash occurred with worst severity:

moderate toxicity, Rash occurred with worst severity: severe toxicity.

- Diarrhea will be analysed patient-based as a categorical variable, with categories: No diarrhea occurred, Diarrhea occurred with worst severity: grade 1, Diarrhea occurred with worst severity: grade 2, Diarrhea occurred with worst severity: grade 3, Diarrhea occurred with worst severity: grade 4 and Diarrhea occurred with worst severity: grade 5.

Observations

	Baseline*	Tarceva® treament follow-up period			Tarceva® treatment stop°	PD after Tarceva®°	End of study°
		FU1	FU2	FU3…			
Informed Consent	X						
Eligibility criteria	X						
Demographic characteristics	X						
Disease characteristics	X						
Previous first-line treatment data	X						
Tarceva® treatment start date and start dose	X						
Tarceva® treatment follow-up dose	(X)	X	X	X			
Tarceva® treatment stop date and reason for stop					X		
Tumor assessments		According to routine clinical practice	According to routine clinical practice	According to routine clinical practice		X (PD)§	
AEs (non-serious and serious)§	(X)	(X)	(X)	(X)	(X)	(X)	(X)
Rash§	(X)	X	X	X	X	X	X
Diarrhea§	(X)	X	X	X	X	X	X
Death (date and reason), Withdrawal of consent (date), Lost-to-follow-up (date) or Study termination (date) (whichever occurs first)							X§

All data collected in this non-interventional study are available from routine clinical assessment and Tarceva® treatment. The protocol does not foresee any mandatory visits schedule or investigations. However all data obtained in routine practice should be captured as long as they fit in the schedule of observations. (FU: follow-up)

* First treatment administration can occur at the most 4 weeks prior to baseline (date of signature of informed consent). If the patient has started treatment with Tarceva® prior to baseline, it is possible that Tarceva® treatment follow-up dose, rash and diarrhea assessment and AEs can already be filled in at baseline.

° Four scenarios are possible: either Tarceva® treatment is stopped due to PD or Tarceva® treatment is stopped before PD, due to toxicity (2 different scenarios) or Tarceva® treatment is stopped due to death, withdrawal of consent, lost-to-follow-up or study termination. Therefore, it is possible that at one timepoint, several of the three columns can be filled in. The study is terminated at one year after the last patient in, or when all enrolled patients have ended the study, whichever occurs first.

§ For safety documentation on the CRF; see section 5: Non-serious AEs (within the period of Tarceva® treatment or 1 month after stop of Tarceva® treatment, before or during the study, except for rash, diarrhea and PD after Tarceva®) and serious AEs (within the period of Tarceva® treatment or 1 month after stop of Tarceva® treatment, before or during the study, except for rash, diarrhea, PD after Tarceva® and death) are documented on an AE page of the CRF. Rash and diarrhea are documented on a separate section of the CRF. PD after Tarceva® is documented on a PD section of the CRF. Death is documented on a study end section of the CRF.
For safety reporting; see section 6: Non-serious AEs (within the period of Tarceva® treatment or 1 month after stop of Tarceva® treatment, during the study, except for rash, diarrhea and PD after Tarceva®) are documented on an AE page of the CRF, will be collected by a monitor and forwarded to Roche. Serious AEs (within the period of Tarceva® treatment or 1 month after stop of Tarceva® treatment, even if the study has been closed, except for PD after Tarceva® and death due to PD) must be reported by the participating physician or designee to Roche within 24 hours, preferably using the AE reporting form.

TABLE OF CONTENTS

1. Background and Rationale ... 15
1.1. Disease Background ... 15
1.2. Drug Background ... 15
1.3. Rationale for the Study and Study Design ... 17
2. Objectives of the Study ... 19
2.1. Primary Objective ... 19
2.2. Secondary Objectives ... 19
3. Study Design ... 21
3.1. Overview of Study Design ... 21
3.2. Study Duration ... 21
3.3. Number of Patients and Centres in the study ... 21
4. Study Population ... 22
4.1. Observed Population ... 22
4.2. Inclusion Criteria for Observation ... 22
4.3. Exclusion Criteria for Observation ... 22
4.4. Tarceva® Treatment Recommendations ... 23
4.5. Concomitant Medication ... 23
5. Schedule of Observations ... 24
5.1. Observation scenarios ... 25
5.1.1. Observation scenario 1 ... 25
5.1.2. Observation scenario 2 ... 26
5.1.3. Observation scenario 3 ... 26
5.1.4. Observation scenario 4 ... 27
5.2. Clinical and Laboratory Observations ... 27
5.2.1. Baseline information ... 27
5.2.2. Tarceva® treatment follow-up information ... 29
5.2.3. Tarceva® treatment stop information ... 30
5.2.4. Information on PD after Tarceva® treatment ... 31
5.2.5. End of study information ... 32
5.3. Criteria for Premature Withdrawals ... 33
6. Adverse Events (AEs) ... 33
6.1. Definition of AEs ... 33
6.1.1. Definition of Serious AEs ... 34
6.1.2. Drug-Adverse Event Relationship ... 34
6.1.3. Causal Attribution Guidance ... 34
6.1.4. Treatment and Follow-up of AEs ... 35

6.1.5. Laboratory Test Abnormalities ... 35
6.1.6. Follow-up of Abnormal Laboratory Test Values ... 35
6.2. Reporting Requirements ... 35
6.2.1. Reporting of Serious AEs ... 35
6.2.2. Non-Serious Adverse Event Reporting ... 36
6.2.3. Non-Serious Adverse Events of Special Interest ... 36
6.2.4. Pregnancy ... 36
6.2.5. Adverse Event Reporting in Final Study Report ... 37
7. Study Termination ... 37
8. Statistical Considerations and Analytical Plan ... 37
8.1. Variables ... 37
8.1.1. Primary Variables ... 37
8.1.2. Secondary Variables ... 37
8.2. Statistical and Analytical Methods ... 39
8.2.1. Statistical Model and Analysis ... 39
8.2.2. Analyses populations ... 39
8.3. Sample Size ... 40
9. Data collection, Management and Quality Assurance ... 40
10. Bibliographic References ... 42
11. Ethical Aspects ... 44
11.1. Guidelines for Non-interventional Studies ... 44
11.2. Informed Consent ... 44
11.3. Regulatory Considerations ... 44
12. Conditions For Modifying The Protocol ... 44
13. Study Documentation, CRFs, Record Keeping and monitoring ... 45
13.1. Investigator's Files/Retention of Documents ... 45
13.2. Monitoring ... 45
13.3. Case Report Forms (CRFs) ... 45
14. Confidentiality of Trial Documents and Patient Records ... 45
15. Agreements ... 45
15.1. Financial Agreement ... 45
15.2. Insurance ... 45
16. Document Retention and Archiving ... 46
17. Resources to conduct the study ... 46
18. Publication of Data ... 46

Glossary Of Abbreviations

AE	adverse event
BSA	body surface area
BSC	best supportive care
CI	confidence interval
CPH	cox proportional hazards
CR	complete response
CRF	case report form
CTCAE	common terminology criteria for adverse events
DCR	disease control rate
DMP	data management plan
DVP	data validation plan
ECOG	Eastern cooperative oncology group
EGFR	epidermal growth factor receptor
FU	follow-up
ICH-GCP	The International Conference on Harmonisation of Technical Requirements for Registration of Pharmaceuticals for Human Use-Good Clinical Practice
GPP	Good Pharmacoepidemiology Practices
GQ	guided questionnaire
HER1	human epidermal growth factor receptor type 1
HR	hazard ratio
ILD	interstitial lung disease
ITT	intent-to-treat
LFU	lost-to-follow-up
NCIC	National Cancer Institute of Canada
NSCLC	non-small cell lung cancer
OR	odd's ratio
OS	overall survival

Glossary Of Abbreviations

PD	progressive disease
PFS	progression-free survival
PP	per protocol
PR	partial response
PS	performance status
PQ	protocol qualified
QoL	quality of life
RECIST	response evaluation criteria in solid tumors
RR	response rate
SA	safety analysis
SAE	serious adverse event
SAP	statistical analysis plan
SD	stabile disease
SDV	source data verification
SmPC	summary of product characteristics
term	study termination
TKI	tyrosine kinase inhibitor
TNM	tumor node metastasis
TOX	toxicity
WC	withdrawal of consent

1. Background and Rationale

1.1. Disease Background

Locally advanced or metastatic non-small cell lung cancer (NSCLC), which accounts for about 80% of all lung cancers, remains largely fatal, with a limited positive impact of chemotherapy due to intrinsic and acquired resistance, manifested clinically by early progression and transient responses. Classical chemotherapy regimens have limited efficacy, and have reached a therapeutic plateau of modest overall survival (OS) benefit (about 8 months up to 10.3 months [1-4]). Even for pemetrexed in combination with cisplatin, the median survival still remains less than one year [5,6]. For these platinum-containing doublets, this and the problem of significant systemic toxicity, such as haematologic toxicity [1] mean that there remains an unmet need for more efficacious agents with reduced toxicity, particularly in the second- and third-line setting, following the completion of aggressive first-line chemotherapy regimens.

1.2. Drug Background

Tarceva® (erlotinib), a potent oral tyrosine kinase inhibitor (TKI) targeting the Human Epidermal Growth Factor Receptor Type 1 (HER1)/Epidermal Growth Factor Receptor (EGFR) is approved for treatment of locally advanced or metastatic NSCLC after failure of at least one prior chemotherapy regimen [7-10]. In Belgium, Tarceva is reimbursed for treatment of locally advanced or metastatic NSCLC after failure of at least one prior chemotherapy regimen, in patients with a tumor that shows EGFR overexpression [11]. Since 2010, it is also approved for maintenance treatment in patients with locally advanced or metastatic NSCLC with stable disease after 4 cycles of standard platinum-based first-line chemotherapy [7]. In Belgium, Tarceva® is reimbursed for maintenance treatment of patients with locally advanced or metastatic NSCLC that show no progressive disease after 4 cycles of standard platinum-based first-line chemotherapy, and with a tumor that contains an activating mutation of EGFR [11].

Tarceva®'s efficacy and survival benefit have been demonstrated in the pivotal National Cancer Institute of Canada (NCIC) BR.21 clinical trial in which 731 patients with stage IIIb/IV NSCLC, who had failed at least one previous chemotherapy regimen, were randomized to receive double-blind 150 mg daily Tarceva® (N=488) or placebo (N=243) [12]. This pivotal phase III trial showed that Tarceva® is effective in these patients (see Table 1). Only 5% of patients discontinued Tarceva® because of toxic effects, so therefore Tarceva® is also a safe and tolerable treatment in these patients.

Table 1 BR.21 clinical trial efficacy results[12] (HR: hazard ratio; PFS: progression-free survival)

	**Tarceva®**	**Placebo**	
RR	8.9%	<1%	P<0.001
Median response duration	7.9 months	3.7 months	
Median PFS	2.2 months	1.8 months	Adjusted HR 0.61, P<0.001
Median OS	6.7 months	4.7 months	HR 0.70, P<0.001

Other approved second-line treatments with proven survival advantages include docetaxel [13] and pemetrexed [14]. The latter is restricted for use in patients with non-squamous histology due to a deleterious outcome effect in the other histology group [6].

Emerging novel targeted therapies present medical and pneumo-oncologists with evolving treatment options for second-line therapy in which factors for consideration should include efficacy, tolerability, quality of life (QoL) improvement and patient preference.

Tarceva® is distinct from chemotherapies, like docetaxel and pemetrexed, by virtue of its targeted mode of action. It allows for a break in chemotherapy when treatment subsequent to first-line chemotherapy is required, allowing for improvement in symptoms and QoL, as shown in the BR.21 study [15], and, arguably, better tolerance of return to chemotherapy or other best supportive care (BSC) regimens used in third-line treatment.

Tarceva® has also demonstrated consistent efficacy across different patient subgroups with various prognostic factors, including *EGFR* mutation status [16], histology [17], gender [17] and smoking status [17,18]. In Belgium, one of the reimbursement criteria for Tarceva® is the presence of EGFR overexpression, as shown by an immunohistochemical test where at least 10% of the cells react positively [11].

Whilst the BR.21 study demonstrated a clear OS benefit in the Tarceva® group, certain patients were ineligible for inclusion, for example, those with brain metastases [12]. Therefore, questions on the impact of the use of Tarceva® in real-life clinical practice remained.

Subsequently, the global TRUST study [19] was set up. This prospective phase IV trial has confirmed the activity and favourable safety profile of Tarceva® after failure of at

least one prior chemotherapy regimen in unselected patients (see Figure 1). In this study with more than 6000 patients, the response rate (RR) was 13%, the disease-control rate (DCR) was 69%, and median PFS and OS were 3.25 months and 7.9 months, respectively. 12% of patients experienced one or more Tarceva®-related adverse events (AEs), other than prespecified AEs defined in the protocol, and only 4% experienced a Tarceva®-related serious AE (SAE). 71% of patients experienced Tarceva®-related rash; of these, the majority of cases were grade 1 or 2.

Figure 1 OS in BR.21[12] and TRUST[19] study

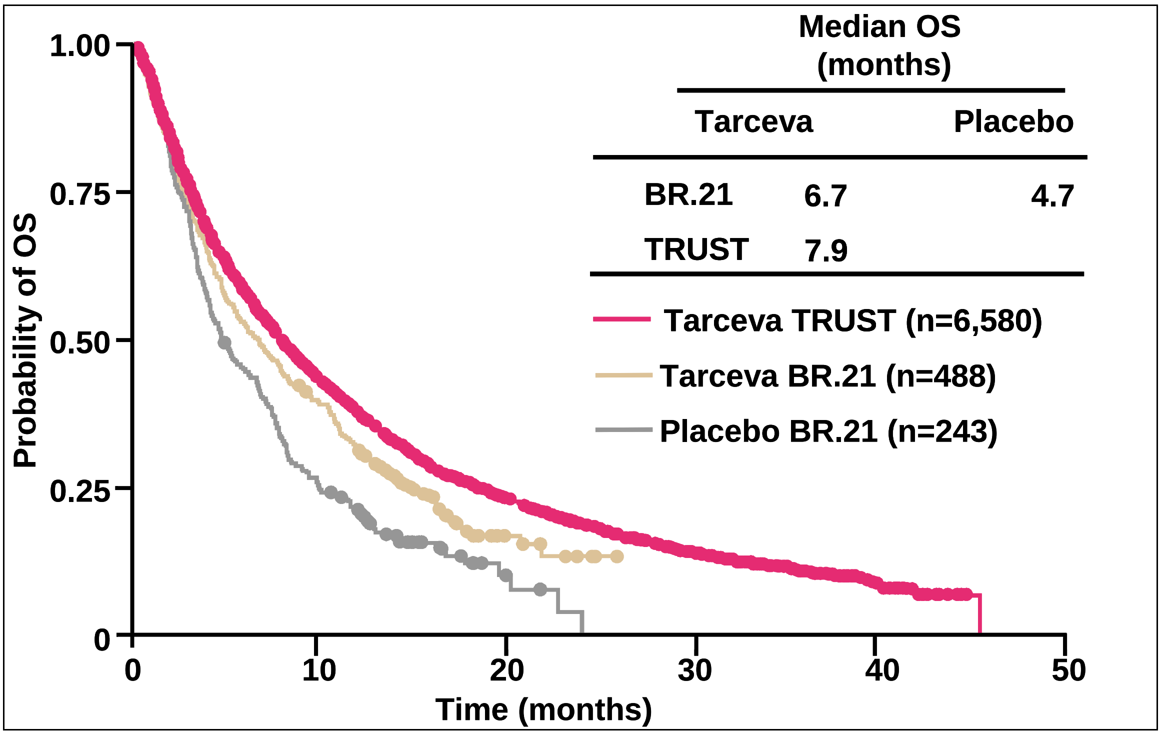

After this, a local prospective non-interventional study TEAM was set up in Belgium and Luxemburg, to observe specificities of NSCLC patients and treatments in a real-life setting in Belgium and Luxemburg. The recruitment for this study has been stopped at the end of 2010, with currently 378 patients and an interim analysis of this study has been reported [20], with confirmed best overall RR of 6.5%, DCR with confirmed responses of 70.7% and median PFS of 2.8 months as preliminary results. No unexpected AEs for Tarceva® were reported. In 50% of patients, rash was reported with the majority of cases grade 1 or 2. The planned final analysis of these data, where also OS and QoL will be assessed, should add to the evolving picture of treatment in practice for NSCLC patients in Belgium and Luxemburg.

1.3. Rationale for the Study and Study Design

In first-line treatment of NSCLC, the number of available treatments is increasing. Besides the reference regimens containing doublet combinations of platinum

compounds with gemcitabine, vinorelbine or taxanes, a new treatment paradigm of first-line treatment with a pemetrexed-containing platinum doublet has been developed, due to the JMDB study [21-23].
This multicentre, randomised, open-label, phase III study of pemetrexed plus cisplatin versus gemcitabine plus cisplatin in chemonaive patients with locally advanced or metastatic NSCLC proved that pemetrexed plus cisplatin (intent-to-treat [ITT] population n = 862) showed similar clinical efficacy as gemcitabine plus cisplatin (ITT n = 863) in OS (adjusted HR 0.94; 95% confidence interval (CI) = 0.84-1.05). This non-inferiority is also supported by the analysis of the protocol qualified (PQ) population.
PFS and overall RR were similar between treatment arms: median PFS was 4.8 months for pemetrexed plus cisplatin versus 5.1 months for gemcitabine plus cisplatin (adjusted HR 1.04; 95% CI = 0.94-1.15), and overall RR was 30.6% (95% CI = 27.3-33.9) for pemetrexed plus cisplatin versus 28.2% (95% CI = 25.0-31.4) for gemcitabine plus cisplatin.
Because of the emerging evidence of a differential expression of thymidilate synthase (a target of pemetrexed) between adenocarcinoma and squamous cell carcinoma, a prespecified subgroup analysis by histology was performed. The analysis of the impact of NSCLC histology on OS demonstrated clinically relevant differences in survival according to histology, see Table 2.

Table 2 JMDB clinical trial efficacy results[21-23]

ITT population and histology subgroups	Median OS in months (95% CI)				Adjusted HR (95% CI)	Superiority p-value
	pemetrexed + cisplatin		gemcitabine + cisplatin			
ITT population (N = 1725)	10.3 (9.8 – 11.2)	N=862	10.3 (9.6 – 10.9)	N=863	0.94 (0.84 – 1.05)	0.259
Adenocarcinoma (N=847)	12.6 (10.7 – 13.6)	N=436	10.9 (10.2 – 11.9)	N=411	0.84 (0.71–0.99)	0.033
Large cell (N=153)	10.4 (8.6 – 14.1)	N=76	6.7 (5.5 – 9.0)	N=77	0.67 (0.48–0.96)	0.027
Other (N=252)	8.6 (6.8 – 10.2)	N=106	9.2 (8.1 – 10.6)	N=146	1.08 (0.81–1.45)	0.586
Squamous cell (N=473)	9.4 (8.4 – 10.2)	N=244	10.8 (9.5 – 12.1)	N=229	1.23 (1.00–1.51)	0.050

As a result of this study, pemetrexed was approved in Europe [7], Canada, and the U.S. in combination with cisplatin for the frontline treatment of nonsquamous NSCLC.

Since May 1, 2009, pemetrexed is reimbursed as first-line treatment of advanced NSCLC in Belgium (in association with cisplatin for NSCLC with non-predominantly squamous histology) [11].

In second-line treatment of NSCLC, Tarceva has been shown to be effective and safe/tolerable (described in the pivotal study BR.21 and confirmed in the global phase IV TRUST and the local phase IV TEAM study.

It has been described that the choice of first-line treatment can have an impact on the effectiveness of second-line treatment [24]. Therefore, a therapeutic question that remains concerns the effectiveness and safety/tolerability of Tarceva® as second-line treatment of advanced NSCLC, after failure of the recently approved pemetrexed-containing chemotherapy regimen as first-line therapy. The current TIME study will describe these aspects of Tarceva® in this setting.

Moreover, some interesting aspects will be described for the first time to our knowledge, like the potential impact of tumor assessments through response evaluation criteria in solid tumors (RECIST) on PFS under Tarceva®, the effectiveness of Tarceva® according to response to first-line treatment with a pemetrexed-containing chemotherapy or according to whether or not presence of brain metastases, and also the incidence and severity of diarrhea will be specifically assessed in Belgium.

In Belgium, lung cancer has a relatively high disease burden, with an incidence of $35.5/10^5$ and a mortality of $29.1/10^5$ [25]. In men, lung cancer is the second most frequent cancer type with an incidence of $57/10^5$ and it is the principal cause of cancer death with a mortality of $50.4/10^5$. In females, lung cancer is the third most frequent cancer type with an incidence of $17.5/10^5$ and it is the second most frequent cause of cancer death with a mortality of $11.8/10^5$.

In Belgium, the real-life clinical data on treatment of patients with NSCLC are limited. Therefore, it would be very useful to conduct this non-interventional study in order to have a view on the Belgian specificities in the management of NSCLC patients undergoing second-line treatment with Tarceva®, after failure of first-line treatment with a pemetrexed-containing chemotherapy regimen.

The aim of the study is to assess the effectiveness and safety/tolerability of Tarceva® and to assess actual day to day disease or patient's management by the physician.

2. Objectives of the Study

2.1. Primary Objective

The primary objective of this study is:

The PFS in patients with advanced stage III/IV NSCLC under Tarceva® as second-line therapy, after failure of a prior pemetrexed-containing chemotherapy regimen, will be described.

2.2. Secondary Objectives

Secondary objectives of this study are:

- The best overall response, best confirmed overall response in case of a best overall response of complete response (CR) or partial response (PR), RR and DCR in patients with advanced stage III/IV NSCLC under Tarceva® as second-line therapy,

after failure of a prior pemetrexed-containing chemotherapy regimen, will be described.

- The OS in patients with advanced stage III/IV NSCLC with Tarceva® as second-line therapy, after failure of a prior pemetrexed-containing chemotherapy regimen, will be described.
- Effectiveness data (PFS under Tarceva®, RR under Tarceva®, DCR under Tarceva® and OS from start of Tarceva® treatment) can be described and compared for different subgroups, depending on the size of the subgroups:

  - subgroup comparison of patients whose tumor response was assessed according to RECIST criteria versus patients whose tumor response was assessed according to other criteria.

  - subgroup comparison of 3 groups: patients whose best overall response to 1L is CR or PR (at baseline) versus patients whose best overall response to 1L is SD (stabile disease) (at baseline) versus patients whose best overall response to 1L is progressive disease (PD) (at baseline).

  - subgroup comparison of 2 groups: patients whose best overall response to 1L is CR or PR or SD (at baseline) versus patients whose best overall response to 1L is PD (at baseline).

  - subgroup comparison of females (at baseline) versus males (at baseline).

  - subgroup comparison of 2 groups: patients whose ECOG performance status (PS) is 0 or 1 (at baseline) versus patients whose ECOG PS is 2 (at baseline).

  - subgroup comparison of 3 groups: patients whose ECOG PS is 0 (at baseline) versus patients whose ECOG PS is 1 (at baseline) versus patients whose ECOG PS is 2 (at baseline).

  - subgroup comparison of patients with brain metastases (at baseline) versus patients with no brain metastases [(stage III cancer stage or bone metastases or liver metastases or adrenal gland metastases or other metastases at baseline) and (no brain metastases at baseline)].

  - subgroup comparison of non-smokers (at baseline) versus former/current smokers (at baseline).

  - subgroup comparison of patients with age <65 years (at baseline) versus patients with age ≥65 years (at baseline).

  - subgroup comparison of patients with no rash during Tarceva® treatment versus patients with rash any toxicity (mild, moderate or severe) during Tarceva® treatment.

  - supportive and additional evaluations may be conducted in other subgroup populations for exploratory reasons. These subgroups will be defined in the statistical analysis plan (SAP).
- Safety and tolerability of Tarceva® as second-line therapy, after failure of a prior pemetrexed-containing chemotherapy regimen, in patients with advanced stage III/IV NSCLC will be described (rash, diarrhea and other AEs).

3. Study Design

3.1. Overview of Study Design

This is a prospective, open-label, multicenter, national, non-interventional phase IV trial of the effectiveness and safety/tolerability of Tarceva® as second-line treatment of patients with advanced NSCLC, after failure of first-line treatment with a pemetrexed-containing chemotherapy regimen. The overall goal is to monitor the “real-life” use of Tarceva® as second-line treatment in patients with advanced NSCLC, after failure of first-line treatment with a pemetrexed-containing chemotherapy regimen, in terms of treatment patterns and associated effectiveness. Another goal is to contribute to the continued pharmacovigilance of Tarceva® in the treatment of advanced NSCLC. Therefore, a prospective, non-interventional, open-label design without treatment randomization is appropriate. Recruiting patients from multiple academic and non-academic centers will mirror the “real-life” distribution of treatment resources for NSCLC patients.

This is a non-interventional study. Patients are started on treatment with Tarceva® per their prescribing physician's best clinical judgement. They are screened for eligibility for the study, and if eligible, they can read and sign the informed consent form. After that, baseline characteristics are assessed. Dosing and treatment duration are at the discretion of the investigator and in accordance with local labeling [summary of product characteristics (SmPC), which can be found at http://www.ema.europa.eu]. The number and frequency of follow-up visits under Tarceva® are according to routine institutional clinical practice. The study is ended for the patients if death, withdrawal of consent, lost-to-follow-up or study termination occurs (whichever occurs first). All assessments are according to routine institutional clinical practice. Only data collected as a part of an investigator's routine clinical practice are to be recorded. Being a non-interventional study, there are no required tests, examinations, or other observations and assessments to be performed. However all data obtained in routine practice should be captured as long as they fit in the schedule of observations.

3.2. Study Duration

The planned study start date (first inclusion) is foreseen in approximately July, 2011. The inclusion period will end on September 30, 2012 and the study participation per patient will be from the first date of Tarceva® treatment until death, withdrawal of consent, lost-to-follow-up or study termination occurs (whichever occurs first).

The study is terminated at one year after the last patient in, or when all enrolled patients have ended the study, whichever occurs first (the rationale for this is discussed in further detail in section 3.3). Termination of the study is thus foreseen in approximately end of September, 2013.

3.3. Number of Patients and Centres in the study

Approximately 20 centres will be involved in the study; both academic and non-

academic. Each individual centre is expected to collect data of approximately 2-3 patients. The study aims to collect data of approximately 55 patients in total.

The sample size for this study was not based on a formal sample size calculation, since the study does not address a specific statistical hypothesis. However, based on literature and previous experience, a sample size of at least 50 patients seems enough to do descriptive analysis. Considering that PFS is expected to be roughly 3 months, a follow-up period of maximum 12 months after conclusion of recruitment should provide sufficiently precise estimates of the primary endpoint PFS and other relevant analysis variables. The subgroup analyses are discussed in further detail in section 8.

4. Study Population

4.1. Observed Population

The observed population consists of patients with advanced NSCLC (inoperable stage III or stage IV) who are treated with Tarceva® as second-line therapy and who have progressed after first-line treatment with a pemetrexed-containing chemotherapy regimen.

Every screened patient must fulfill the eligibility criteria (see inclusion- and exclusion criteria) to be considered eligible for participation.

4.2. Inclusion Criteria for Observation

Patients can be entered in the study if:

- The patients signed the written informed consent.
- The patients are ≥18 years.
- The patients have had a histologically or cytologically documented diagnosis of locally advanced or metastatic NSCLC (inoperable stage III or IV according to 7th Tumor Node Metastasis (TNM) classification (see appendix 1)).
- The patients have an ECOG PS of 0-2.
- The patients have been experiencing PD after pemetrexed-containing chemotherapy regimen as first-line chemotherapy.
- The patients have started treatment with Tarceva® as 2L therapy, at the most 4 weeks prior to study entry at baseline (date of signature of informed consent).

4.3. Exclusion Criteria for Observation

Patients can not be entered in the study if:

- The patients received already other chemotherapy/targeted therapy treatment after PD after 1L treatment in advanced NSCLC setting.
- The patients have a contra-indication as in SmPC of Tarceva®: *Hypersensitivity to Tarceva® or to any of the excipients.*

4.4. Tarceva® Treatment Recommendations

Dosing, treatment duration, dose modifications, interruptions, and delays of Tarceva® are per the prescribing physician's best clinical judgement and in accordance with local labeling (SmPC):

- The recommended daily dose of Tarceva® is 150 mg taken at least one hour before or two hours after the ingestion of food.

- Use of Tarceva® in patients with severe hepatic dysfunction is not recommended.

- Use of Tarceva® in patients with severe renal impairment is not recommended.

- The Tarceva® tablets contain lactose and should not be administered to patients with rare hereditary problems of galactose intolerance, Lapp lactase deficiency or glucose-galactose malabsorption.

- Women of childbearing potential must be advised to avoid pregnancy while on Tarceva®. Adequate contraceptive methods should be used during therapy, and for at least 2 weeks after completing therapy. Treatment should only be continued in pregnant women if the potential benefit to the mother outweighs the risk to the foetus.

- Because of the potential harm to the infant, mothers should be advised against breast-feeding while receiving Tarceva®.

- Current smokers should be advised to stop smoking, as plasma concentrations of Tarceva® in smokers as compared to non-smokers are reduced.

- Concomitant use of CYP3A4 substrates and modulators may require dose adjustment.

- When dose adjustment is necessary, the dose should be reduced in 50mg steps.

4.5. Concomitant Medication

All concomitant medications as used in daily routine clinical practice and not contraindicated in the local label (SmPC) for Tarceva® are allowed, but will not be reported in the case report form (CRF).

5. Schedule of Observations

	Baseline*	Tarceva® treament follow-up period			Tarceva® treatment stop°	PD after Tarceva®°	End of study°
		FU1	FU2	FU3…			
Informed Consent	X						
Eligibility criteria	X						
Demographic characteristics	X						
Disease characteristics	X						
Previous first-line treatment data	X						
Tarceva® treatment start date and start dose	X						
Tarceva® treatment follow-up dose	(X)	X	X	X			
Tarceva® treatment stop date and reason for stop					X		
Tumor assessments		According to routine clinical practice	According to routine clinical practice	According to routine clinical practice		X (PD)[§]	
AEs (non-serious and serious)[§]	(X)	(X)	(X)	(X)	(X)	(X)	(X)
Rash[§]	(X)	X	X	X	X	X	X
Diarrhea[§]	(X)	X	X	X	X	X	X
Death (date and reason), Withdrawal of consent (date), Lost-to-follow-up (date) or Study termination (date) (whichever occurs first)							X[§]

All data collected in this non-interventional study are available from routine clinical assessment and Tarceva® treatment. The protocol does not foresee any mandatory visits schedule or investigations. However all data obtained in routine practice should be captured as long as they fit in the schedule of observations. (FU: follow-up)

* First treatment administration can occur at the most 4 weeks prior to baseline (date of signature of informed consent). If the patient has started treatment with Tarceva® prior to baseline, it is possible that Tarceva® treatment follow-up dose, rash and diarrhea assessment and AEs can already be filled in at baseline.

° Four scenarios are possible: either Tarceva® treatment is stopped due to PD or Tarceva® treatment is stopped before PD, due to toxicity (2 different scenarios) or Tarceva® treatment is stopped due to death, withdrawal of consent, lost-to-follow-up or study termination. Therefore, it is possible that at one timepoint, several of the three columns can be filled in. The study is terminated at one year after the last patient in, or when all enrolled patients have ended the study, whichever occurs first.

[§] For safety documentation on the CRF; see section 5: Non-serious AEs (within the period of Tarceva® treatment or 1 month after stop of Tarceva® treatment, before or during the study, except for rash, diarrhea and PD after Tarceva®) and serious AEs (within the period of Tarceva® treatment or 1 month after stop of Tarceva® treatment, before or during the study, except for rash, diarrhea, PD after Tarceva® and death) are documented on an AE page of the CRF. Rash and diarrhea are documented on a separate section of the CRF. PD after Tarceva® is documented on a PD section of the CRF. Death is documented on a study end section of the CRF.
For safety reporting; see section 6: Non-serious AEs (within the period of Tarceva® treatment or 1 month after stop of Tarceva® treatment, during the study, except for rash, diarrhea and PD after Tarceva®) are documented on an AE page of the CRF, will be collected by a monitor and forwarded to Roche. Serious AEs (within the period of Tarceva® treatment or 1 month after stop of Tarceva® treatment, even if the study has been closed, except for PD after Tarceva® and death due to PD) must be reported by the participating physician or designee to Roche within 24 hours, preferably using the AE reporting form.

5.1. Observation scenarios

As schematically depicted in the table on the previous page, the patient is observed during his/her second-line treatment with Tarceva®. However, the study also collects data about the Tarceva® treatment stop, PD after Tarceva® and the end of the study. It is possible that several of these 3 elements coincide, as illustrated in the 4 figures underneath representing the 4 possible scenarios.

The study is terminated at one year after the last patient in, or when all enrolled patients have ended the study, whichever occurs first.

5.1.1. Observation scenario 1

Stop of Tarceva® treatment due to progressive disease. Death, withdrawal of consent (WC), lost-to-follow-up (LFU) or study termination (term) occurs later and this means the end of the study (see Figure 2).

Figure 2 Observation scenario 1

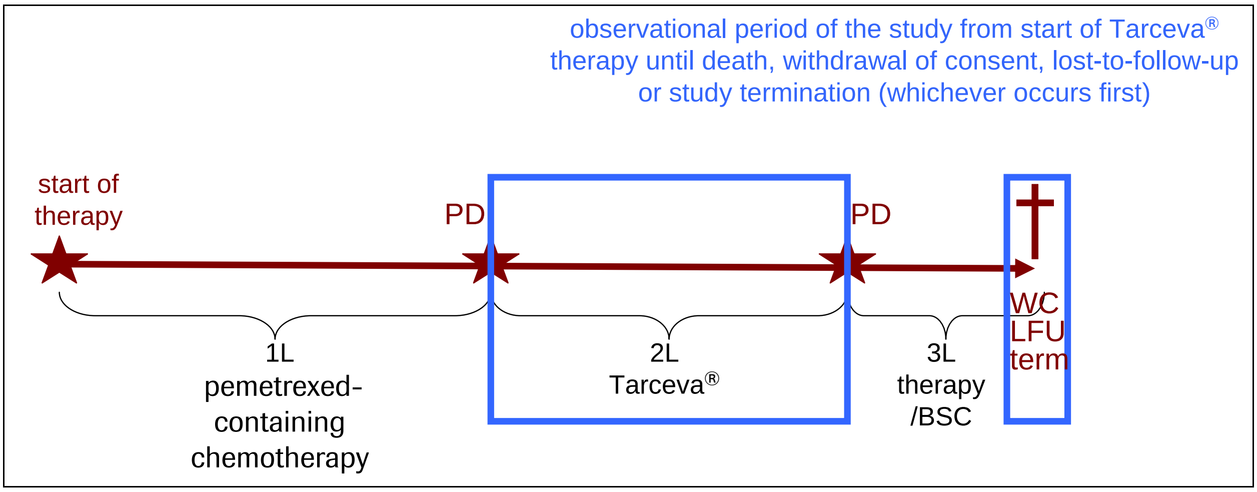

5.1.2. Observation scenario 2

Stop of Tarceva® treatment due to intolerability/unacceptable toxicity (TOX). Progressive disease after Tarceva® treatment occurs later. Death, withdrawal of consent, lost-to-follow-up or study termination occurs even later and this means the end of the study (see Figure 3).

Figure 3 Observation scenario 2

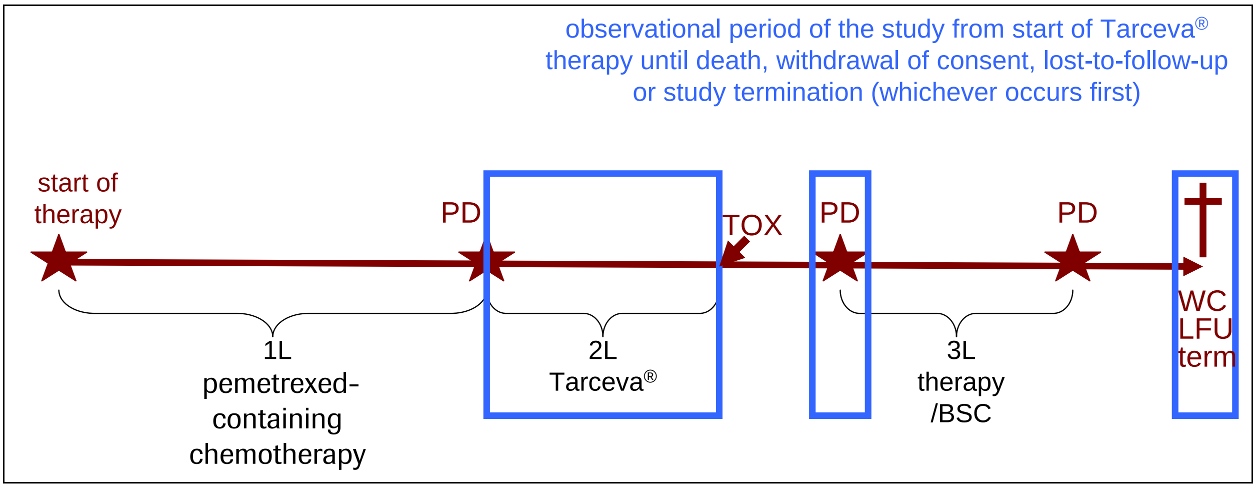

5.1.3. Observation scenario 3

Stop of Tarceva® treatment due to intolerability/unacceptable toxicity. Death, withdrawal of consent, lost-to-follow-up or study termination occurs later and this means the end of the study (see Figure 4).

Figure 4 Observation scenario 3

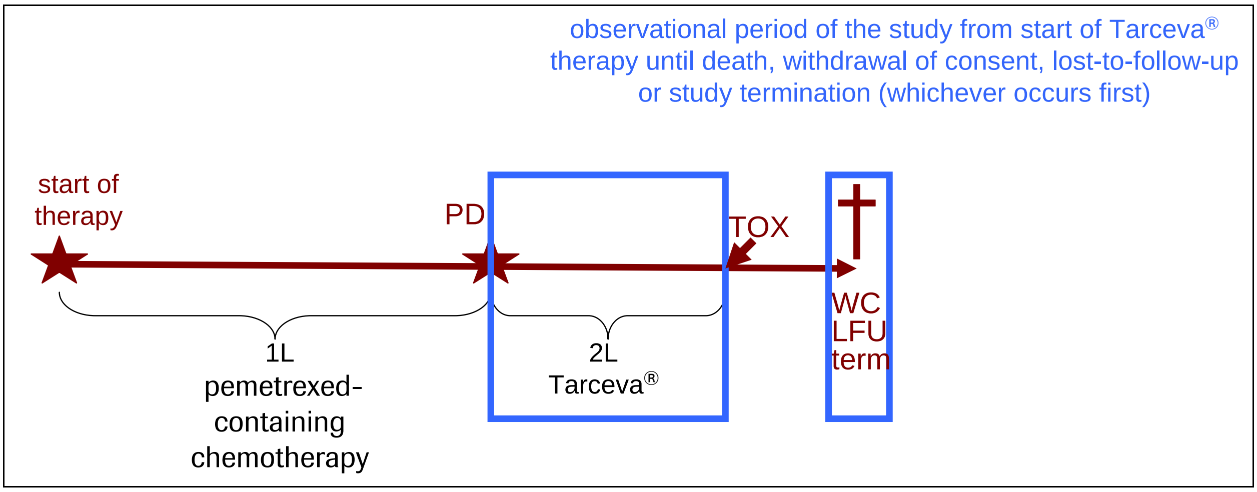

5.1.4. Observation scenario 4

Stop of Tarceva® treatment due to death, withdrawal of consent, lost-to-follow-up or study termination and this means the end of the study (see Figure 5).

Figure 5 Observation scenario 4

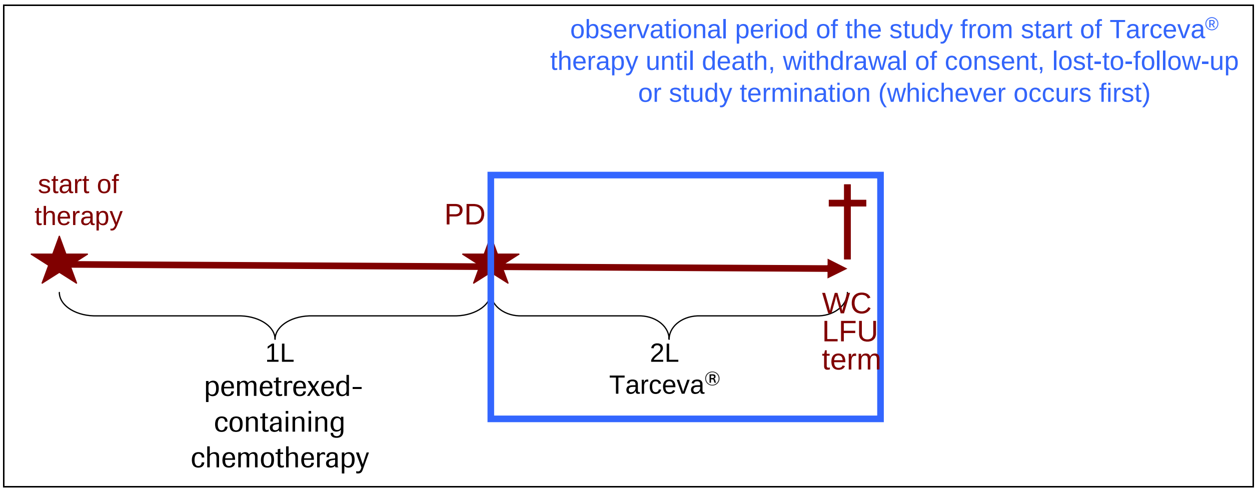

5.2. Clinical and Laboratory Observations

In the routine clinical care setting, patient with advanced NSCLC are seen regularly by their treating physicians, either for treatment or for regular assessments during/after treatment. Therefore, no study-specific visits or evaluations are required per protocol. However all data obtained in routine practice should be captured as long as they fit in the schedule of observations.

All clinical and laboratory information collected during this non-interventional study is routinely documented in the patient's medical records. It will be extracted from the patient's medical records and entered into the CRF.

5.2.1. Baseline information

- First of all, the patient is screened if he/she meets the **eligibility criteria** (see inclusion- and exclusion criteria) to be considered eligible for participation. If eligible for participation, a **signed written informed consent** is asked. A copy of the signed informed consent must be given to the patient.

- Patient's **demographic characteristics** (date of birth, gender and ethnic origin, smoking status (for the definition; see appendix 2) will be extracted from the medical record and entered into the CRF, if available.

- Patient's **disease characteristics** prior to Tarceva® treatment [current ECOG PS score (see appendix 3), date of initial diagnosis of NSCLC, current cancer stage according to 7th TNM classification (see appendix 1), tumor histology, metastasis localization, tumor specimen type

(biopsy/cytology), EGFR immunohistochemistry status, EGFR mutation status] will be extracted from the medical record and entered into the CRF, if available.

- Patient's **previous first-line treatment** (type, dose and regimen, start and stop date of 1L treament, reason for stop of 1L treatment, best overall response under 1L treatment, progression date after 1L treatment) will be extracted from the medical record and entered into the CRF, if available. The best overall response is determined according to routine institutional clinical practice: through RECIST criteria or through other criteria.

- Patient's **treatment start date and dose of Tarceva®** will be extracted from the medical record and entered into the CRF. First treatment administration can occur at the most 4 weeks prior to Baseline (date of signature of informed consent).

- If the patient has started treatment with Tarceva® prior to study entry at baseline (date of signature of informed consent), it is possible that Tarceva® treatment follow-up dose, rash and diarrhea assessment and AEs can already be filled in at baseline.

- Patient's **treatment follow-up dose of Tarceva®** will be extracted from the medical record and entered into the CRF. Temporary treatment interruption has to be mentioned in the CRF but is not considered as treatment stop if the Tarceva® treatment is restarted and no other chemotherapy/targeted therapy has been used during the interruption.

- Patient's **non-serious AEs** (other than rash, diarrhea and PD after Tarceva®) that occur within the period of Tarceva® treatment or 1 month after stop of Tarceva® treatment, before or during the study, will be extracted from the medical record and entered on an AE page of the CRF.

- Patient's **rash assessment** (serious or non-serious) that occurs within the period of Tarceva® treatment or 1 month after stop of Tarceva® treatment, before or during the study, is done according to Thatcher *et al.* (see appendix 5) and is documented on a separate section of the CRF.

- Patient's **diarrhea assessment** (serious or non-serious) that occurs within the period of Tarceva® treatment or 1 month after stop of Tarceva® treatment, before or during the study, is done according to CTCAE (version 4.0; see appendix 6) and is documented on a separate section of the CRF.

- Patient's **SAEs** (other than rash, diarrhea, PD after Tarceva® and death) that occur within the period of Tarceva® treatment or 1 month after stop of Tarceva® treatment, before or during the study, will be extracted from the medical record and entered on an AE page of the CRF.

For serious and all other AEs, the following must be assessed and recorded on the AE page of the CRF: name of reporter, seriousness, start and stop date, intensity, relationship to Tarceva®, action taken regarding Tarceva®, treatment given for the event

and outcome.

- The **reporting of safety/tolerability data**; is described in detail in section 6 and summarized as follows. Non-serious AEs (other than rash, diarrhea and PD after Tarceva®), that occur within the period of Tarceva® treatment or 1 month after stop of Tarceva® treatment, during the study, are documented on an AE page of the CRF, will be collected by a monitor and forwarded to Roche. Serious AEs (other than PD after Tarceva® and death due to PD), that occur within the period of Tarceva® treatment or 1 month after stop of Tarceva® treatment, even if the study has been closed, must be reported by the participating physician or designee to Roche within 24 hours, preferably using the AE reporting form.

5.2.2. Tarceva® treatment follow-up information

- Patient's **treatment follow-up dose of Tarceva®** will be extracted from the medical record and entered into the CRF. Temporary treatment interruption has to be mentioned in the CRF but is not considered as treatment stop if the Tarceva® treatment is restarted and no other chemotherapy/targeted therapy has been used during the interruption.

- Patient's **tumor assessment** during Tarceva® treatment, with frequency, method and criteria according to routine institutional clinical practice: radiographic assessment [through RECIST criteria (version 1.1; see appendix 4) or through other interpretation criteria (non-standardized, non-quantitative criteria)] or other assessment (e.g. clinical criteria like palpation of superficial lymph nodes, or appearance of visible cutaneous metastasis) will be extracted from the medical record and entered into the CRF, if available. Also, the **method and criteria for tumor assessment** [radiographic assessment [through RECIST criteria (version 1.1; see appendix 4) or through other interpretation criteria (non-standardized, non-quantitative criteria)] or other assessment (e.g. clinical criteria like palpation of superficial lymph nodes, or appearance of visible cutaneous metastasis)] will be extracted from the medical record and entered into the CRF, if available.

- Patient's **non-serious AEs** (other than rash, diarrhea and PD after Tarceva®) that occur within the period of Tarceva® treatment or 1 month after stop of Tarceva® treatment, before or during the study, will be extracted from the medical record and entered on an AE page of the CRF.

- Patient's **rash assessment** (serious or non-serious) that occurs within the period of Tarceva® treatment or 1 month after stop of Tarceva® treatment, before or during the study, is done according to Thatcher *et al.* (see appendix 5) and is documented on a separate section of the CRF.

- Patient's **diarrhea assessment** (serious or non-serious) that occurs within the period of Tarceva® treatment or 1 month after stop of Tarceva® treatment, before or during the study, is done according to CTCAE (version 4.0; see appendix 6) and is documented on a separate section of the CRF.

- Patient's **SAEs** (other than rash, diarrhea, PD after Tarceva® and death) that occur within the period of Tarceva® treatment or 1 month after stop of Tarceva® treatment, before or during the

study, will be extracted from the medical record and entered on an AE page of the CRF.

For serious and all other AEs, the following must be assessed and recorded on the AE page of the CRF: name of reporter, seriousness, start and stop date, intensity, relationship to Tarceva®, action taken regarding Tarceva®, treatment given for the event and outcome.

- The **reporting of safety/tolerability data**; is described in detail in section 6 and summarized as follows. Non-serious AEs (other than rash, diarrhea and PD after Tarceva®), that occur within the period of Tarceva® treatment or 1 month after stop of Tarceva® treatment, during the study, are documented on an AE page of the CRF, will be collected by a monitor and forwarded to Roche. Serious AEs (other than PD after Tarceva® and death due to PD), that occur within the period of Tarceva® treatment or 1 month after stop of Tarceva® treatment, even if the study has been closed, must be reported by the participating physician or designee to Roche within 24 hours, preferably using the AE reporting form.

5.2.3. Tarceva® treatment stop information

- Patient's **Tarceva® treatment stop date and the reason for stop** will be extracted from the medical record and entered into the CRF. Temporary treatment interruption is not considered as treatment stop if the Tarceva® treatment is restarted and no other chemotherapy/targeted therapy has been used during the interruption.

- Patient's **non-serious AEs** (other than rash, diarrhea and PD after Tarceva®) that occur within the period of Tarceva® treatment or 1 month after stop of Tarceva® treatment, before or during the study, will be extracted from the medical record and entered on an AE page of the CRF.

- Patient's **rash assessment** (serious or non-serious) that occurs within the period of Tarceva® treatment or 1 month after stop of Tarceva® treatment, before or during the study, is done according to Thatcher *et al.* (see appendix 5) and is documented on a separate section of the CRF.

- Patient's **diarrhea assessment** (serious or non-serious) that occurs within the period of Tarceva® treatment or 1 month after stop of Tarceva® treatment, before or during the study, is done according to CTCAE (version 4.0; see appendix 6) and is documented on a separate section of the CRF.

- Patient's **SAEs** (other than rash, diarrhea, PD after Tarceva® and death) that occur within the period of Tarceva® treatment or 1 month after stop of Tarceva® treatment, before or during the study, will be extracted from the medical record and entered on an AE page of the CRF.

For serious and all other AEs, the following must be assessed and recorded on the AE page of the CRF: name of reporter, seriousness, start and stop date, intensity, relationship to Tarceva®, action taken regarding Tarceva®, treatment given for the event and outcome.

- The **reporting of safety/tolerability data**; is described in detail in section 6 and summarized as follows. Non-serious AEs (other than rash, diarrhea and PD after Tarceva®), that occur

within the period of Tarceva® treatment or 1 month after stop of Tarceva® treatment, during the study, are documented on an AE page of the CRF, will be collected by a monitor and forwarded to Roche. Serious AEs (other than PD after Tarceva® and death due to PD), that occur within the period of Tarceva® treatment or 1 month after stop of Tarceva® treatment, even if the study has been closed, must be reported by the participating physician or designee to Roche within 24 hours, preferably using the AE reporting form.

5.2.4. Information on PD after Tarceva® treatment

- **Date of patient's progressive disease after Tarceva® treatment** will be extracted from the medical record and entered on a PD section of the CRF. Also, the **method and criteria for tumor assessment** of PD [radiographic assessment [through RECIST criteria (version 1.1; see appendix 4) or through other interpretation criteria (non-standardized, non-quantitative criteria)] or other assessment (e.g. clinical criteria like palpation of superficial lymph nodes, or appearance of visible cutaneous metastasis)] will be extracted from the medical record and entered on a PD section of the CRF, if available.

- **No reporting of PD** to Roche is required.

- Patient's **non-serious AEs** (other than rash, diarrhea and PD after Tarceva®) that occur within the period of Tarceva® treatment or 1 month after stop of Tarceva® treatment, before or during the study, will be extracted from the medical record and entered on an AE page of the CRF.

- Patient's **rash assessment** (serious or non-serious) that occurs within the period of Tarceva® treatment or 1 month after stop of Tarceva® treatment, before or during the study, is done according to Thatcher *et al.* (see appendix 5) and is documented on a separate section of the CRF.

- Patient's **diarrhea assessment** (serious or non-serious) that occurs within the period of Tarceva® treatment or 1 month after stop of Tarceva® treatment, before or during the study, is done according to CTCAE (version 4.0; see appendix 6) and is documented on a separate section of the CRF.

- Patient's **SAEs** (other than rash, diarrhea, PD after Tarceva® and death) that occur within the period of Tarceva® treatment or 1 month after stop of Tarceva® treatment, before or during the study, will be extracted from the medical record and entered on an AE page of the CRF.

For serious and all other AEs, the following must be assessed and recorded on the AE page of the CRF: name of reporter, seriousness, start and stop date, intensity, relationship to Tarceva®, action taken regarding Tarceva®, treatment given for the event and outcome.

- The **reporting of safety/tolerability data**; is described in detail in section 6 and summarized as follows. Non-serious AEs (other than rash, diarrhea and PD after Tarceva®), that occur within the period of Tarceva® treatment or 1 month after stop of Tarceva® treatment, during the study, are documented on an AE page of the CRF, will be collected by a monitor and forwarded to Roche. Serious AEs (other than PD after Tarceva® and death due to PD), that occur within

the period of Tarceva® treatment or 1 month after stop of Tarceva® treatment, even if the study has been closed, must be reported by the participating physician or designee to Roche within 24 hours, preferably using the AE reporting form.

5.2.5. End of study information

There are 4 possible events that lead to end of study for the patient: death, withdrawal of consent, lost-to-follow-up or study termination. The one that is applicable is the one that occurs first.

- **Date of patient's death and the reason** will be extracted from the medical record and entered on a study end section of the CRF.

- **No reporting of death due to PD** to Roche is required. **If the reason for death is not PD, the event must be reported to Roche with death as outcome**, if it occurs within the period of Tarceva® treatment or 1 month after stop of Tarceva® treatment, even if the study has been closed. It must be reported by the participating physician or designee to Roche within 24 hours, preferably using the AE reporting form.

- **Date of patient's withdrawal of consent** will be extracted from the medical record and entered on a study end section of the CRF.

- **Date of patient's lost-to-follow-up** will be extracted from the medical record and entered on a study end section of the CRF. It is preferred that the prescribing physician or an assigned person has tried to contact the patient or his/her general physician at least once.

- **Date of study termination**. If none of the 3 above events has occurred and one year after the last patient in has passed, the study is terminated. In this case, this means the end of study for the patient. The date of study termination will be communicated by Roche (estimated approximately end of September, 2013) and entered on a study end section of the CRF.

- Patient's **non-serious AEs** (other than rash, diarrhea and PD after Tarceva®) that occur within the period of Tarceva® treatment or 1 month after stop of Tarceva® treatment, before or during the study, will be extracted from the medical record and entered on an AE page of the CRF.

- Patient's **rash assessment** (serious or non-serious) that occurs within the period of Tarceva® treatment or 1 month after stop of Tarceva® treatment, before or during the study, is done according to Thatcher *et al.* (see appendix 5) and is documented on a separate section of the CRF.

- Patient's **diarrhea assessment** (serious or non-serious) that occurs within the period of Tarceva® treatment or 1 month after stop of Tarceva® treatment, before or during the study, is done according to CTCAE (version 4.0; see appendix 6) and is documented on a separate section of the CRF.

- Patient's **SAEs** (other than rash, diarrhea, PD after Tarceva® and death) that occur within the period of Tarceva® treatment or 1 month after stop of Tarceva® treatment, before or during the study, will be extracted from the medical record and entered on an AE page of the CRF.

For serious and all other AEs, the following must be assessed and recorded on the AE page of the CRF: name of reporter, seriousness, start and stop date, intensity, relationship to Tarceva®, action taken regarding Tarceva®, treatment given for the event and outcome.

- The **reporting of safety/tolerability data**; is described in detail in section 6 and summarized as follows. Non-serious AEs (other than rash, diarrhea and PD after Tarceva®), that occur within the period of Tarceva® treatment or 1 month after stop of Tarceva® treatment, during the study, are documented on an AE page of the CRF, will be collected by a monitor and forwarded to Roche. Serious AEs (other than PD after Tarceva® and death due to PD), that occur within the period of Tarceva® treatment or 1 month after stop of Tarceva® treatment, even if the study has been closed, must be reported by the participating physician or designee to Roche within 24 hours, preferably using the AE reporting form.

5.3. Criteria for Premature Withdrawals

The patient, prescribing physician and NV Roche SA have the right to prematurely withdraw the patient from the study at any time; e.g. in case of a screening failure or protocol violation. Screening failures are defined as patients who are screened at baseline, but are found by the investigator not to be eligible for participation in the study (see inclusion- and exclusion criteria). No other information except the screening information is desired for these patients. Protocol violators are defined as patients who are screened at baseline, and found by the investigator to be eligible for participation in the study. Further information for these patients is collected, but at a certain point during the study or even after the study end for these patients, they are found to be violators of the study protocol.

6. Adverse Events (AEs)

6.1. Definition of AEs

According to the The International Conference on Harmonisation of Technical Requirements for Registration of Pharmaceuticals for Human Use-Good Clinical Practice (ICH-GCP) guidelines: an AE is any untoward medical occurrence in a subject or clinical investigation subject administered a pharmaceutical product and which does not necessarily have a causal relationship with this treatment. An AE can therefore be any unfavorable and unintended sign (including an abnormal laboratory finding); symptom; or disease temporally associated with the use of a medicinal product, whether or not consider related to the medicinal product.

All clinical AEs encountered within the period of Tarceva® treatment or 1 month after stop of Tarceva® treatment, will be reported on the AE page of the CRF.

Intensity of AEs will be graded on a three-point scale (mild, moderate, severe) and reported in detail on the CRF.

<u>Definition of AE intensity scale:</u>

Mild	Discomfort noticed but no disruption of normal daily activity
Moderate	Discomfort sufficient to reduce or affect daily activity

Severe Inability to work or perform normal daily activity

Rash and diarrhea will be graded on a different scale, according to the toxicity definition by Thatcher *et al.* and CTCAE version 4.0, respectively; see appendix 5 and 6, respectively). For PD after Tarceva® and death, no grading will be applied.

6.1.1. Definition of Serious AEs

A SAE is any experience that suggests a significant hazard, contraindication, side effect or precaution. It is an AE that at any dose fulfils at least one of the following criteria:

- Is fatal (results in death);

  Note: death is an outcome, not an event
- Is Life-Threatening

  Note: the term 'life-threatening' refers to an event in which the subject was at immediate risk of death at the time of the event; it does not refer to an event which could hypothetically have caused a death had it been more severe
- Required in-subject hospitalization or prolongation of existing hospitalization
- Results in persistent or significant disability / incapacity
- Is a congenital anomaly / birth defect
- Is medically significant or requires intervention to prevent one or other of the outcomes listed above

The term "sudden" should only be used when the cause is of a cardiac origin as per standard definition. The terms "death" and "sudden death" are clearly distinct and must not be used interchangeably.

All cases of suspected transmission of an infectious agent via medicinal product and all pregnancy reports should be handled as "serious" (reported by the participating physician or designee to Roche within 24 hours, preferably using the AE Reporting Form).

6.1.2. Drug-Adverse Event Relationship

Relationship of the AE to the treatment should always be assessed by the investigator. The causality relationship of Tarceva® to the AE will be assessed by the investigator as either **YES** or **NO.** To ensure consistency of causality assessments, investigators should apply the following general guidance.

6.1.3. Causal Attribution Guidance

Is there a reasonable possibility that the Roche drug Tarceva® caused the AE based on facts (evidence) or arguments to suggest a causal relationship?

YES
(The AE is related to Roche drug Tarceva®)
If there is a plausible temporal relationship between the onset of the AE and administration of the Roche drug, and the AE cannot be readily explained by the subject's clinical state, intercurrent illness, or concomitant therapies: and/or the AE follows a known pattern of response to the Roche drug; and/or the AE abates or resolves upon discontinuation of the Roche drug or dose reduction and, if applicable, reappears upon re-challenge.

NO
(The AE is not related to Roche drug Tarceva®)
If clear evidence exists that the AE has an etiology other than the Roche drug (e.g., preexisting medical condition, underlying disease, intercurrent illness, or concomitant medication): and/or the AE has no plausible temporal relationship to administration of the study drug (e.g., cancer diagnosed 2 days after first dose of Roche drug.

6.1.4. Treatment and Follow-up of AEs

AEs, especially those for which the relationship to Roche drug Tarceva® is "related", should be followed up until they have returned to baseline status or stabilized. If after follow-up, return to baseline status or stabilization cannot be established "ongoing at the end of the study" should be recorded on the CRF. Patients experiencing AEs should be treated by accepted clinical procedures.

6.1.5. Laboratory Test Abnormalities

Any laboratory result abnormality fulfilling the criteria for a SAE should be reported as such, in addition to being recorded on an AE page of the CRF.

Any treatment-emergent abnormal laboratory result which is clinically significant, i.e., meeting one or more of the following conditions, should be recorded as a single diagnosis on an AE page in the CRF:

- Accompanied by clinical symptoms;
- Leading to a change in Roche drug Tarceva® (e.g. dose modification, interruption or permanent discontinuation);
- Requiring a change in concomitant therapy (e.g. addition of, interruption of, discontinuation of, or any other change in a concomitant medication, therapy or treatment).

6.1.6. Follow-up of Abnormal Laboratory Test Values

At decision of investigator and according to routine daily practice.

6.2. Reporting Requirements

6.2.1. Reporting of Serious AEs

Any clinical AE or abnormal laboratory test value that is ***serious*** [as defined in section 6.1.1 above] and which occurs within the period of Tarceva® treatment or 1 month after stop of

Tarceva® treatment, even if the study has been closed, must be reported to Roche within ***24 hours*** of the investigator becoming aware of the event. The only exceptions are PD after Tarceva® and death due to PD (deemed to be consistent with the natural course of disease), for which the data from the clinical database (see section 5.2) will be transferred to the safety database at the termination of the study, for the final study report. The SAE is reported by the site by means of fax, email or phone but preferably using the AE Reporting Form.

The term "severe" is a measure of intensity, thus a severe AE is not necessarily serious. For example, nausea of several hours' duration may be rated as severe, but may not be clinically serious.

For serious and all other AEs, the following must be assessed and recorded on the AE page of the CRF: name of reporter, seriousness, start and stop date, intensity, relationship to Tarceva®, action taken regarding Tarceva®, treatment given for the event and outcome.

6.2.2. Non-Serious Adverse Event Reporting

Non-serious AEs which occur within the period of Tarceva® treatment or 1 month after stop of Tarceva® treatment, during the study, are documented on an AE page of the CRF, will be collected by a monitor and forwarded to Roche. The only exceptions are PD after Tarceva® (deemed to be consistent with the natural course of disease), rash and diarrhea (most common side-effects of Tarceva®), for which the data from the clinical database (see section 5.2) will be transferred to the safety database at the termination of the study, for the final study report.

6.2.3. Non-Serious Adverse Events of Special Interest

AEs of special interest are defined in the latest version of the risk management plan of Tarceva®. Currently, these are: drug-induced liver injury, hearing loss and tinnitus, interstitial lung disease (ILD) and thrombotic microangiopathy, but these might change over time.

Non-serious AEs of special interest which occur within the period of Tarceva® treatment or 1 month after stop of Tarceva® treatment, during the study,should be handled as "serious" (reported by the participating physician or designee to Roche within 24 hours, preferably using the AE Reporting Form).

In order to obtain more information on AEs of special interest, a Guided Questionnaire (GQ) may be provided to the investigator. The investigator or designee is requested to fill out the GQ appropriately and return it to Roche within reasonable timelines.

6.2.4. Pregnancy

A female patient must be instructed to stop taking the Roche drug Tarceva® and immediately inform the investigator if she becomes pregnant during the study. The investigator should report all pregnancies within 24 hours of knowledge to the sponsor on the *Pregnancy Reporting Form, gcp_for000084*. The investigator should counsel the patient; discuss the risks of continuing with the pregnancy and the possible effects on the foetus, as per the SmPC: *Women of childbearing potential must be advised to avoid pregnancy while on Tarceva®. Adequate contraceptive methods should be used during therapy, and for at least 2 weeks after completing therapy.*

Treatment should only be continued in pregnant women if the potential benefit to the mother outweighs the risk to the foetus.

Monitoring of the patient should continue until conclusion of the pregnancy. If clinically necessary, follow-up may continue for a period of time after birth of child. All pregnancies must be followed up until the outcome is known irrespective of date of database closure / study completion.

Pregnancy occurring in the partner of a male patient participating in the study should be reported to the investigator and the sponsor. The partner should be counseled, the risks of continuing the pregnancy discussed, as well as the possible effects on the foetus. Monitoring of the patient should continue until conclusion of the pregnancy. Only if the pregnant partner has signed a *Pregnant Partner Data Release Form, gcp_for000186*, a *Pregnancy Reporting Form, gcp_for000084* should be filled out by the investigator.

6.2.5. Adverse Event Reporting in Final Study Report

The collected AEs from all sites are assessed for potential signals by the responsible person at the local Pharmacovigilance department on an ongoing basis and compiled in the Final Study Report.
The only exceptions are non-serious rash and diarrhea (most common side-effects of Tarceva®), PD after Tarceva® and death due to PD (both deemed to be consistent with the natural course of disease), for which the data from the clinical database (see section 5.2) will be transferred to the safety database at the termination of the study, for the final study report.

7. Study Termination

The study is terminated at one year after the last patient in, or when all enrolled patients have ended the study, whichever occurs first. Termination of the study is thus foreseen in approximately end of September, 2013.

8. Statistical Considerations and Analytical Plan

8.1. Variables

8.1.1. Primary Variables

- The PFS in patients with advanced stage III/IV NSCLC under Tarceva® as second-line therapy, after failure of a prior pemetrexed-containing chemotherapy regimen, will be described.

    This variable will be analysed by the Kaplan-Meier method. Kaplan-Meier estimates for the survival rate and a Kaplan-Meier curve for survival will be presented. If possible the median survival time will be reported.

8.1.2. Secondary Variables

- The OS in patients with advanced stage III/IV NSCLC with Tarceva® as second-line therapy, after failure of a prior pemetrexed-containing chemotherapy regimen, will be described.

    This variable will be analysed by the Kaplan-Meier method. Kaplan-Meier estimates for the survival rate and a Kaplan-Meier curve for survival will be presented. If possible the

median survival time will be reported.

- The best overall response under Tarceva®, best confirmed overall response under Tarceva® in case of a best overall response of CR or PR, RR under Tarceva® and DCR under Tarceva® in patients with advanced stage III/IV NSCLC under Tarceva® as second-line therapy, after failure of a prior pemetrexed-containing chemotherapy regimen, will be described through descriptive statistics.

    - Best overall response under Tarceva® is defined as the best tumor assessment during Tarceva® treatment (according to RECIST criteria or through other criteria), with hierarchy of CR>PR>SD>PD.

    - Best confirmed overall response under Tarceva® will be described in case of a best overall response of CR or PR. Best confirmed overall response is defined as CR if at least two tumor assessments of CR have been made during Tarceva® treatment. Best confirmed overall response is defined as PR, either if one tumor assessment of CR and at least one of PR have been made, or if at least two tumor assessments of PR have been made during Tarceva® treatment. Best confirmed overall response is defined as SD, if the definitions above of best confirmed overall response of CR or PR do not apply and at least one tumor assessment of SD has been made during Tarceva® treatment. Best confirmed overall response is defined as PD, if the definitions above of best confirmed overall response of CR or PR or SD do not apply and at least one tumor assessment of PD has been made during Tarceva® treatment.

    - RR under Tarceva® is defined as the rate of CR or PR as best overall response under Tarceva®.

    - DCR under Tarceva® is defined as the rate of CR or PR or SD as best overall response under Tarceva®.

- Subgroup analyses can be made to describe possible relationships between baseline and other assessments and the outcomes PFS, OS, RR and DCR, depending on the size of the subgroups.

    - The subgroup analyses on time-to-event variables (PFS and OS) will be graphically presented by Kaplan-Meier curves, if possible, where p-values will be the results of a Log-Rank test and Wilcoxon test. In case both test results differ substantially, the Log-Rank test will be used for interpretation. The results of these tests can be further investigated by Cox Proportional Hazards models (CPH), to give a quantitative measurement for difference, such as a HR and its 95% confidence limits. A p-value for the test of statistical significance of that HR will be presented. The underlying assumption on proportional hazards of the CPH model will be tested.

    - The subgroup analyses for binary data (RR and DCR) will be performed using a logistic regression model, if possible, resulting in odd's ratio (OR) estimates with their 95% confidence limits. A p-value for statistical significance of this OR will be presented.

    - The subgroups are defined in section 8.2.2.2.

- Safety and tolerability of Tarceva® in 2L will be described through descriptive statistics:

    - AEs will be analysed patient-based, with categories: treatment-emergent AEs, post-treatment AEs.

    - SAEs will be analysed patient-based.

- Deaths will be analysed patient-based.

- Rash will be analysed patient-based as a categorical variable, with categories: No rash occurred, Rash occurred with worst severity: mild toxicity, Rash occurred with worst severity: moderate toxicity and Rash occurred with worst severity: severe toxicity.

- Diarrhea will be analysed patient-based as a categorical variable, with categories: No diarrhea occurred, Diarrhea occurred with worst severity: grade 1, Diarrhea occurred with worst severity: grade 2, Diarrhea occurred with worst severity: grade 3, Diarrhea occurred with worst severity: grade 4 and Diarrhea occurred with worst severity: grade 5.

8.2. Statistical and Analytical Methods

8.2.1. Statistical Model and Analysis

This non-interventional, exploratory, non-comparative study aims primarily to describe PFS and other variables rather than to test pre-specified statistical hypotheses. Statistical analyses will be performed primarily for descriptive and estimation purposes. However, statistical models and tests may be used to explore interesting aspects of the data. Unless otherwise specified, all statistical hypotheses will be tested at the 5% significance level against two-sided alternatives, and corresponding 95% confidence intervals will be reported as appropriate. There will be no correction for multiplicity due to the explorative nature of analyses.

8.2.2. Analyses populations

8.2.2.1. Safety analysis population (SA) and Per-protocol (PP) population

Safety analyses will be performed only on the SA population, which is all patients included in the study with at least one intake of study medication (Start date of therapy with Tarceva® documented), i.e. screened patients minus screening failures.

All other analyses (except for demographic parameters) will be done only on the PP population, which is all patients of the safety analysis population except for patients with protocol violations, i.e. screened patients minus screening failures minus protocol violators.

Demographic parameters will be analysed for both populations.

8.2.2.2. Subgroup populations

Effectiveness data (PFS under Tarceva®, RR under Tarceva®, DCR under Tarceva® and OS from start of Tarceva® treatment) can be described and compared for different subgroups, depending on the size of the subgroups:

- subgroup comparison of patients whose tumor response was assessed according to RECIST criteria versus patients whose tumor response was assessed according to other criteria.
- subgroup comparison of 3 groups: patients whose best overall response to 1L is CR or PR (at baseline) versus patients whose best overall response to 1L is SD (at baseline) versus patients whose best overall response to 1L is PD (at baseline).

- subgroup comparison of 2 groups: patients whose best overall response to 1L is CR or PR or SD (at baseline) versus patients whose best overall response to 1L is PD (at baseline).

- subgroup comparison of females (at baseline) versus males (at baseline).

- subgroup comparison of 2 groups: patients whose ECOG PS is 0 or 1 (at baseline) versus patients whose ECOG PS is 2 (at baseline).

- subgroup comparison of 3 groups: patients whose ECOG PS is 0 (at baseline) versus patients whose ECOG PS is 1 (at baseline) versus patients whose ECOG PS is 2 (at baseline).

- subgroup comparison of patients with brain metastases (at baseline) versus patients with no brain metastases [(stage III cancer stage or bone metastases or liver metastases or adrenal gland metastases or other metastases at baseline) and (no brain metastases at baseline)].

- subgroup comparison of non-smokers (at baseline) versus former/current smokers (at baseline).

- subgroup comparison of patients with age <65 years (at baseline) versus patients with age ≥65 years (at baseline).

- subgroup comparison of patients with no rash during Tarceva® treatment versus patients with rash any toxicity (mild, moderate or severe) during Tarceva® treatment.
- supportive and additional evaluations may be conducted in other subgroup populations for exploratory reasons. These subgroups will be defined in the SAP.

8.3. Sample Size

The sample size for this study was not based on a formal sample size calculation, since the study does not address a specific statistical hypothesis. However, based on literature and previous experience, a sample size of at least 50 patients is enough to do a descriptive analysis. Considering that PFS is expected to be roughly 3 months, a follow-up period of maximum 12 months after conclusion of recruitment should provide sufficiently precise estimates of the primary endpoint PFS and other relevant analysis variables. The subgroup analyses have been discussed in the text above.

.

9. Data collection, Management and Quality Assurance

<u>Methods used in assembling the study data</u>:

For each patient enrolled, a paper CRF must be completed and signed by the principal investigator or authorized delegate from the study staff. This also applies to records for those patients who fail to complete the study. If a patient withdraws from the study, the reason must be noted on the end-of-study section of the CRF. Data from the source documents will be transcribed by the investigator or a delegate in the CRF.

Dedicated Roche monitors will visit the centre approximately 2 times per year in order to perform a verification and cross–check of the CRFs against the investigator’s records (source data verification: SDV) following good pharmacoepidemiology practices (GPP) Guidelines. SDV may be conducted in 100% of observed patients in each participating center. Before the monitoring visits all patients' performed visits should be completed in the CRFs. During these

monitoring visits the monitor should have direct access to the patients' medical files to perform SDV. CRFs will be collected by the monitor during the monitoring visits.

Roche will send the collected CRFs to the data management vendor by courier service on a regular base.

Procedures for data management:

A data management plan (DMP) will be generated by the data management vendor and approved by Roche. It will contain all necessary elements, according to the the Roche Standard Operational Procedures.

A database will be created and maintained by a dedicated data management vendor. Data will be entered in a database by a dedicated data management vendor. A single data entry will be performed for all CRFs. Coding of the AEs will be done by the data management vendor (as agreed between the data management vendor and Roche).

An annotated CRF will be prepared by the data management vendor. This document will contain the names of tables and variables. Each variable will be associated with its type, its length and any format. It will be submitted for approval to Roche. Before entering real data, the database structure and input screens will be tested and validated in accordance with Standard Operating Procedures of the data management vendor. For this, fictitious CRFs (usually 2), will be completed and processed. The final structure of the database will be submitted for approval to Roche before entering real data.

Also, a data validation plan (DVP) will be prepared by the data management. This document will contain automatic checks (eg consistency checks to detect inconsistencies) and checks by medical review by the data management vendor that will be run on a regular basis. To obtain a clean database (before database lock) the investigator or delegate will be requested to answer, sign and date the CRF specific queries. These controls will be programmed by the data management vendor and then tested with dummy data. These fictitious data and documentation related to testing will be documented by the data management vendor and available for review by Roche. The data validation process will run continuously, a specific request for each inconsistency will be electronically processed by the data management vendor. The queries (listed by site) will be sent to ROCHE by the data management vendor for review by e-mail and then sent to the investigator by the Roche monitor. The queries solved by the investigators will be sent back to ROCHE or collected by the Roche monitor. The answered query will be forwarded per courier to the data management vendor to update the database. Original answered queries need to be collected by the Roche monitor. To limit the number of queries to submit to participating physicians, a study guide of obvious corrections (Self Evident Correction) prepared by the data management vendor and validated by Roche may be realized. Periodic reports of data validation will be edited by the data management vendor and sent to Roche.

The database lock will be realized by the data management vendor after approval by Roche and after the data entry, data control, data cleaning and coding. When the database lock has been achieved, the database will be made available for statistical analysis. The database will be transferred to the statistics vendor. After project completion, the database will be transferred to Roche on CD-ROM.

Methods for data analysis:

The cleaned data will be analyzed by a dedicated statistics vendor, according to the SAP (as described in section 8). A report of the statistics will be made by the statistics vendor. This can serve as a preparation for the final study report and possibly publications on the study.

A description of quality assurance and quality control procedures for all phases of the study:
The overall procedures for quality assurance of clinical study data are described in the Roche Standard Operational Procedures. Quality assurance will also be assured by data management vendor, as described in the DMP.

10. Bibliographic References

[1] Schiller JH, Harrington D, Belani, CP, Langer C, Sandler A, Krook J, Zhu J, Johnson DH, for the Eastern Cooperative Oncology Group. Comparison of four chemotherapy regimens for advanced non-small-cell lung cancer. N Engl J Med 2002; 346(2):92-98.

[2] Kelly K, Crowley J, Bunn PA, Presant CA, Grevstad PK, Moinpour CM,Ramsey SD, Wozniak AJ, Weiss GR, Moore DF, Israel VK, Livingston RB, Gandara DR. Randomized phase III trial of paclitaxel plus carboplatin versus vinorelbine plus cisplatin in the treatment of patients with advanced non–small-cell lung cancer: A Southwest Oncology Group trial. J Clin Oncol 2001; 19(13):3210-18.

[3] Higgins MJ, Ettinger JS. Chemotherapy for lung cancer: The state of the art in 2009. Expert Rev Anticancer Ther 2009; 9(10):1365-78.

[4] Non-Small Cell Lung Cancer Collaborative Group. Chemotherapy and supportive care versus supportive care alone for advanced non-small cell lung cancer. Cochrane Database of Systematic Reviews 2010, Issue 5. Art. No.: CD007309. DOI:10.1002/14651858.CD007309.pub2.

[5] Scagliotti GV, Parikh P, von Pawel J, Biesma B, Vansteenkiste J, Manegold C, Serwatowski P, Gatzemeier U, Digumarti R, Zukin M, Lee JS, Mellemgaard A, Park K, Patil S, Rolski J, Goksel T, de Marinis F, Simms L, Sugarman KP, Gandara D. Phase III study comparing cisplatin plus gemcitabine with cisplatin plus pemetrexed in chemotherapy-naive patients with advanced-stage non-small-cell lung cancer. J Clin Oncol 2008; 26(21):3543-51.

[6] Scagliotti GV, Hanna N, Fosella F et al. The differential efficacy of pemetrexed according to NSCLC histology: a review of two Phase III studies. Oncologist 2009; 14(3):253-63.

[7] http://www.ema.europa.eu.

[8] National Comprehensive Cancer network (NCCN) Clinical practice guidelines in oncology. Non-small cell lung cancer. V.1. 2011.

[9] Azzoli CG, Baker S Jr, Temin S, Pao W, Aliff T, Brahmer J, Johnson DH, Laskin JL, Masters G, Milton D, Nordquist L, Pfister DG, Piantadosi S, Schiller JH, Smith R, Smith TJ, Strawn JR, Trent D, Giaccone G. American Society of Clinical Oncology (ASCO) Clinical Practice Guideline Update on Chemotherapy for Stage IV Non–Small-Cell Lung Cancer. J Clin Oncol 2009; 27(36): 6251-66.

[10] D'Addario G, Früh M, Reck M, Baumann P, Klepetko W, Felip E on behalf of the ESMO Guidelines Working Group. Metastatic non-small-cell lung cancer: ESMO Clinical Practice Guidelines for diagnosis, treatment and followup. Ann Oncol 2010; 21 (Supplement 5): v116–v119.

[11] http://www.riziv.fgov.be.

[12] Shepherd FA, Pereira JR, Ciuleanu T, Tan EH, Hirsh V, Thongprasert S, Campos D, Maoleekoonpiroj S, Smylie M, Martins R, van Kooten M, Dediu M, Findlay B, Tu D, Johnston D, Bezjak A, ClarkG, Santabárbara P, Seymour L for the National Cancer Institute of Canada Clinical Trials Group. Erlotinib in Previously Treated Non–Small-Cell Lung Cancer. N Engl J Med 2005; 353:123-32.

[13] Shepherd FA, Dancey J, Ramlau R, Mattson K, Gralla R, O'Rourke M, Levitan N, Gressot L, Vincent M, Burkes R, Coughlin S, Kim Y, Berille J. Prospective randomized trial of docetaxel versus best supportive care in patients with non–small-cell lung cancer previously treated with platinum-based chemotherapy. J Clin Oncol 2000; 18:2095-103.

[14] Hanna N, Shepherd FA, Fossella FV, Pereira JR, De Marinis F, von Pawel J, Gatzemeier U, Tsao TCY, Pless M, Muller T, Lim H-L, Desch C, Szondy K, Gervais R, Shaharyar, Manegold C, Paul S, Paoletti P, Einhorn L, Bunn Jr. PA. Randomized phase III trial of pemetrexed versus docetaxel in patients with non–small-cell lung cancer previously treated with chemotherapy. J Clin Oncol 2004; 22:1589-97.

[15] Bezjak A, Tu D, Seymour L, Clark G, Trajkovic A, Zukin M, Ayoub J, Lago S, de Albuquerque Ribeiro R, Gerogianni A, Cyjon A, Noble J, Laberge F, Tsz-Tong Chan R, Fenton D, von Pawel J, Reck M, Shepherd FA. Symptom improvement in lung cancer patients treated with erlotinib: quality of life analysis of the National Cancer Institute of Canada Clinical Trials Group Study BR.21. J Clin Oncol 2006; 24:3831-37.

[16] Zhu C-O, da Cunha Santos G, Ding K, Sakurada A, Cutz J-C, Liu N, Zhang T, Marrano P, Whitehead M, Squire JA, Kamel-Reid S, Seymour L, Shepherd FA, Tsao M-S. Role of KRAS and EGFR as biomarkers of response to erlotinib in National Cancer Institute of Canada Clinical Trials Group Study BR.21. J Clin Oncol 2008; 26:4268-75.

[17] Clark GM. Prognostic factors versus predictive factors: Examples from a clinical trial of erlotinib. Molecular Oncol 2008; 1:406-12.

[18] Clark GM, Zborowski DM, Santabarbara P, Ding K, Whitehead M, Seymour L, Shepherd FA. Smoking history and epidermal growth factor receptor expression as predictors of survival benefit from erlotinib for patients with non-small cell lung cancer in the National Cancer Institute of Canada Clinical Trials Group Study BR.21. Clin Lung Cancer 2006; 7(6):389-94.

[19] Reck M, van Zandwijk N, Gridelli C, et al. Erlotinib in advanced Non-small Cell Lung Cancer: Efficacy and Safety Findings of the Global Phase IV Tarceva® Lung Cancer Survival Treatment Study. J Thor Oncol 2010; 5(10):1616-22.

[20] Roche, data on file.

[21] Scagliotti GV, Parikh P, von Pawel J, Biesma B, Vansteenkiste J, Manegold C, Serwatowski P, Gatzemeier U, Digumarti R, Zukin M, Lee JS, Mellemgaard A, Park K, Patil S, Rolski J, de Marinis TF, Simms L, Sugarman KP, Gandara D. Phase III Study Comparing Cisplatin Plus Gemcitabine With Cisplatin Plus Pemetrexed in Chemotherapy-Naïve Patients With Advanced-Stage Non–Small-Cell Lung Cancer. J Clin Oncol 2008; 26(21):3543-3551.

[22] Scagliotti G, Hanna N, Fossella F, Sugarman K, Blatter J, Peterson P, Simms L, Shepherd FA. The Differential Efficacy of Pemetrexed According to NSCLC Histology: A Review of Two Phase III Studies. The Oncologist 2009; 14:253–263.

[23] SmPC Alimta (http://www.ema.europa.eu).

[24] Weiss GJ, Rosell R, Fossella F, Perry M, Stahel R, Barata F, Nguyen B, Paul S, McAndrews P, Hanna N, Kelly K, Bunn PA Jr. The impact of induction chemotherapy on the outcome of second-line therapy with pemetrexed or docetaxel in patients with advanced non-small-cell lung cancer. Ann Oncol. 2007; 18(3):453-60.

[25] GLOBOCAN data (http://www-dep.iarc.fr).

11. Ethical Aspects

11.1. Guidelines for Non-interventional Studies

The guidelines for Good Pharmacoepidemiology Practices (GPP) will be respected as well as recommendations for non-interventional trials and principles of epidemiology studies. This trial is not in the scope of GCP studies.

11.2. Informed Consent

It is the responsibility of the investigator, or a person designated by the investigator, to obtain written informed consent from each individual participating in this study after adequate explanation of the aims, methods, and objectives and potential hazards of the study. It must also be explained to the patients that they are completely free to refuse to enter the study or to withdraw from it at any time for any reason. Patients must agree to the coded data collection, the pooling of data with similar scientific data, and the possibility of monitoring activities between his/her patient file and the CRF by Roche personnel or Roche contracted monitors and regulatory personnel. The patient must receive a copy of the signed informed consent form.

Appropriate forms for patient information and obtaining written informed consent will be provided by the investigator or by Roche/designee. Roche will provide to all investigators a sample patient information form together with a patient consent form.

This protocol and any accompanying material provided to the patient (such as patient information and informed consent sheets) as well as any advertising or compensation given to the patient, will be submitted by the coordinating investigator to an Ethics Committee. Local regulations and requirements will be followed strictly. Approval from the committee must be obtained before starting the study, and should be documented in a letter to the investigator specifying the date on which the committee met and granted the approval. The approval letter must contain the protocol version and date, list all submitted documents (e.g. patient information and consent) including the version number and the composition of the EC members.

Any modifications made to the protocol after receipt of the Ethics Committee approval must also be submitted by the investigator to the Ethics Committee in accordance with local procedures and regulatory requirements.

11.3. Regulatory Considerations

Each participating investigator, in collaboration with the local Roche affiliate is responsible for ensuring that all local regulatory requirements are met prior and during the conduct of this non-interventional trial.

12. Conditions For Modifying The Protocol

Protocol modifications must be made only after consultation between an appropriate representative of the sponsor and the investigator. Protocol modifications must be prepared by a representative of the sponsor and initially reviewed and approved by another competent person of the sponsor.

All protocol modifications must be submitted to the appropriate Ethics Committee for information and approval in accordance with local requirements. Approval must be awaited before any changes can be implemented.

Both the sponsor and the investigator reserve the right to terminate the study at any time. Should this be necessary, both parties will arrange the procedures on an individual study basis after review and consultation. In terminating the study, Roche and the investigator will assure that adequate consideration is given to the protection of the patient's interests.

13. Study Documentation, CRFs, Record Keeping and monitoring

13.1. Investigator's Files/Retention of Documents

The Investigator must maintain adequate and accurate records to enable the conduct of this study.

The Investigator's File will contain the protocol/amendments, Ethics Committee letter and EC vote, sample and signed informed consents as well as other (sample) material given to the patient and a patient enrolment identification log. Signed informed consent forms and the patient enrolment identification log is only part of the investigator's file and must not be made available to Roche. A copy of the signed informed consent must be given to the patient.

13.2. Monitoring

The investigator should understand that source documents for this trial should be made available to appropriately qualified personnel from Roche or Roche designee for monitoring purposes. The monitoring may be conducted for all of the patients in all participating centres.

13.3. Case Report Forms (CRFs)

Data for this study will be captured via a paper CRF.

The investigator should ensure the accuracy, completeness and timelines of the data reported to the sponsor in the CRF and in all required reports.

14. Confidentiality of Trial Documents and Patient Records

The investigator must assure that patients' anonymity will be maintained and that their identities are protected from unauthorized parties. On CRFs or other documents submitted to the sponsor, patients should not be identified by their names, but by an identification code. The investigator should keep a patient enrolment log showing codes, names and addresses. The investigator should maintain documents not for submission to Roche, e.g. patients' written consent forms and the patient enrolment log, in strict confidence.

15. Agreements

15.1. Financial Agreement

The financial agreement between the principal investigator at each site and Roche affiliate for the conduct of this study are separate agreements to the protocol. The investigator must provide details of the study budget/financial agreement to the ethics committee if required.

15.2. Insurance

Roche is responsible for ensuring that adequate insurance is in place to cover the conduct of

this study at each participating site, if appropriate. The specificity of these non-interventional trials means the risk to be run is minimal and reduced to respect of privacy of the trial participants. Roche must be notified immediately of any complaint or claim arising out of or in connection with the study.

16. Document Retention and Archiving

The investigator is responsible for retaining relevant documents in accordance with local and Roche requirements. The investigator is responsible for ensuring that study records are not accidentally destroyed; patients' medical records should be clearly labelled to ensure that they are not accidentally destroyed in error.

Once the study is complete, archiving of the survey documentation may occur away from the investigational site. In this case, Roche must be informed. Documentation showing the date and location of archived documentation must be kept at the local research site in the event that survey documentation needs to be recalled for audit purposes.

The Investigator's File must be archived according to local requirements and for 20 years.

17. Resources to conduct the study

Dedicated Roche monitors will visit the center approximately 2 times per year. Before the monitoring visits all patients' performed visits should be completed in the CRFs. During these monitoring visits the monitor should have direct access to the patients' medical files to perform SDV.

Accurate and reliable data collection will be assured by verification and cross-check of the CRFs against the investigator's records by the study monitor (SDV) following good pharmacoepidemiology practices (GPP) Guidelines. SDV may be conducted in 100% of observed patients in each participating center.

All collected data will be reviewed and managed by a dedicated data management vendor, according to the DMP. To obtain a clean database (before database lock) the investigator or delegate will be requested to answer, sign and date the CRF specific queries (obtained by automatic checks constructed in the database and by medical review by the data management vendor, according to the DVP).

The cleaned data will be analyzed by a dedicated statistics vendor, according to the SAP (as described in section 8).

18. Publication of Data

In accord with standard editorial and ethical practice, Roche will generally support publication of multicenter non-interventional studies only in their entirety and not as individual centre data. In this case, a coordinating investigator will be designated by mutual agreement.

Authorship will be determined by mutual agreement.

There is an ethical obligation to disseminate findings of potential scientific or public health importance (e.g. results pertaining to the safety of a marketed medication).

Appendix 1:

More info on 7th edition of the NSCLC stage classification system:

NSCLC stage classification	
**Stage**	**TNM**
0	TisN0M0
IA	T1aN0M0<br>T1bN0M0
IB	T2aN0M0
IIA	T1aN1M0<br>T1bN1M0<br>T2aN1M0<br>T2bN0M0
IIB	T2bN1M0<br>T3N0M0
IIIA	T1N2M0<br>T2N2M0<br>T3N1M0<br>T3N2M0<br>T4N0M0<br>T4N1M0
IIIB	T4N2M0<br>AnyTN3M0
IV	AnyTAnyNM1a<br>AnyTAnyNM1b

Reference:

Tanoue LT et al. "New TNM classification for non-small cell lung cancer" Expert Rev. Anticancer Ther. 2009; 9: 413-423.

Appendix 2:

More info on smoking status:

Smoking status classification	
**Smoking status**	
Former/Current smoker	The overall lifetime exposure has been more than 100 cigarettes (or equivalent).
Non-smoker	The overall lifetime exposure has been less than or equal to 100 cigarettes (or equivalent).

Reference:

Centers for Disease Control. "Cigarette smoking among adults—United States, 2000". Morbidity and Mortality Weekly Report 2002; 51: 642–645.

Appendix 3:

More info on ECOG performance status:

ECOG PERFORMANCE STATUS	
**Grade**	**ECOG**
0	Fully active, able to carry on all pre-disease performance without restriction.
1	Restricted in physically strenuous activity but ambulatory and able to carry out work of a light or sedentary nature, e.g. light house work, office work.
2	Ambulatory and capable of all selfcare but unable to carry out any work activities. Up and about more than 50% of waking hours.
3	Capable of only limited selfcare, confined to bed or chair more than 50% of waking hours.
4	Completely disabled. Cannot carry on any selfcare. Totally confined to bed or chair.
5	Dead.

Reference:

Oken MM et al. "Toxicity And Response Criteria Of The Eastern Cooperative Oncology Group" American Journal of Clinical Oncology 1982; 5: 649-655.

Appendix 4:

More info on RECIST criteria:

RECIST CRITERIA	
**Tumor assessment**	
CR	Disappearance of all target lesions.
PR	Min. 30% decrease in the sum of diameters of target lesions (ref. the baseline sum of diameters).
SD	Neither sufficient shrinkage to qualify for PR nor sufficient increase to qualify for PD (ref. the smallest sum diameters while on the study).
PD	Min. 20% increase in the sum of diameters of target lesions (ref. the smallest sum of diameters on the study incl. baseline if this is the smallest sum).<br>*Note: appearance of 1 or more new lesions is also considered progression.*

Reference:

Eisenhauer EA et al. "New response evaluation criteria in solid tumours: Revised RECIST guideline (version 1.1)" European Journal of Cancer 2009; 45: 228-247.

Appendix 5:

More info on rash toxicity:

RASH	
**Toxicity**	
Mild	Usually localized.<br>Minimally symptomatic.<br>No impact on activities of daily living.<br>No ulceration, weeping, or infection.
Moderate	Localized or generalized.<br>Mild symptoms (e.g. pruritus, tenderness).<br>Minimal impact on activities of daily living.<br>No ulceration, weeping, or infection.
Severe	Usually generalized.<br>Severe symptoms (e.g. pruritus, tenderness).<br>Significant impact on activities of daily living.<br>Ulceration, weeping, or other evidence of infection present.

Reference:

Thatcher N et al. "Expert Consensus on the Management of Erlotinib-Associated Cuteneous Toxicity in the U.K. (U.K. Erlotinib Skin Toxicity Management Consensus Group grading system)" The Oncologist 2009; 14: 840-847.

Appendix 6:

More info on diarrhea grade:

**DIARRHEA** (Definition: A disorder characterized by frequent and watery bowel movements.)	
**Grade**	
1	Increase of <4 stools per day over baseline; mild increase in ostomy output compared to baseline
2	Increase of 4 - 6 stools per day over baseline; moderate increase in ostomy output compared to baseline
3	Increase of >=7 stools per day over baseline; incontinence; hospitalization indicated; severe increase in ostomy output compared to baseline; limiting self care ADL
4	Life-threatening consequences; urgent intervention indicated
5	Death

Reference:

"Common Terminology Criteria for Adverse Events (CTCAE) version 4.0" (as published on http://ctep.cancer.gov/ on September 15, 2009).
